# HSP90α is needed for the survival of rod photoreceptors and regulates the expression of rod PDE6 subunits

**DOI:** 10.1016/j.jbc.2023.104809

**Published:** 2023-05-11

**Authors:** Daniella Munezero, Hunter Aliff, Ezequiel Salido, Thamaraiselvi Saravanan, Urikhan Sanzhaeva, Tongju Guan, Visvanathan Ramamurthy

**Affiliations:** 1 Department of Pharmaceutical and Pharmacological Sciences, West Virginia University, Morgantown, West Virginia, USA; 2 Ophthalmology and Visual Sciences, West Virginia University, Morgantown, West Virginia, USA; 3 Biochemistry and Molecular Medicine, West Virginia University, Morgantown, West Virginia, USA

**Keywords:** chaperone, heat shock protein 90 (HSP90), cochaperone, cyclic GMP (cGMP), phosphodiesterase 6 (PDE6), photoreceptor, vision, retinal degeneration

## Abstract

Heat shock protein 90 (HSP90) is an abundant molecular chaperone that regulates the stability of a small set of proteins essential in various cellular pathways. Cytosolic HSP90 has two closely related paralogs: HSP90α and HSP90β. Due to the structural and sequence similarities of cytosolic HSP90 paralogs, identifying the unique functions and substrates in the cell remains challenging. In this article, we assessed the role of HSP90α in the retina using a novel HSP90α murine knockout model. Our findings show that HSP90α is essential for rod photoreceptor function but was dispensable in cone photoreceptors. In the absence of HSP90α, photoreceptors developed normally. We observed rod dysfunction in HSP90α knockout at 2 months with the accumulation of vacuolar structures, apoptotic nuclei, and abnormalities in the outer segments. The decline in rod function was accompanied by progressive degeneration of rod photoreceptors that was complete at 6 months. The deterioration in cone function and health was a “bystander effect” that followed the degeneration of rods. Tandem mass tag proteomics showed that HSP90α regulates the expression levels of <1% of the retinal proteome. More importantly, HSP90α was vital in maintaining rod PDE6 and AIPL1 cochaperone levels in rod photoreceptor cells. Interestingly, cone PDE6 levels were unaffected. The robust expression of HSP90β paralog in cones likely compensates for the loss of HSP90α. Overall, our study demonstrated the critical need for HSP90α chaperone in the maintenance of rod photoreceptors and showed potential substrates regulated by HSP90α in the retina.

Heat shock proteins (HSPs) are a group of conserved and abundant proteins that aid in cellular function and survival (1, 2, 3, 4). HSPs are classified based on their molecular weights and include small HSPs, HSP40, HSP60, HSP70, HSP90, and HSP100 (5). HSPs function as molecular chaperones to assist in the folding of nascent proteins, refolding denatured proteins, and degradation of aberrant proteins in the cell (6, 7, 8, 9, 10, 11, 12, 13, 14, 15, 16). Among HSPs, HSP90 binds and folds a selected set of proteins called clients (17). HSP70 partially folds HSP90 clients before their interaction with the HSP90 system (18, 19, 20). A set of proteins known as cochaperones are thought to regulate HSP90 activity and assist in substrate recruitment, thereby determining the specificity of HSP90 clients (21, 22). HSP90 works closely with the proteasomal system to ensure the clearance of aggregated HSP90 clients (23).

HSP90 is a therapeutic target in cancer treatment due to its role in stabilizing oncogenic clients and acting as a biochemical buffer to maintain proteins necessary for tumor proliferation (24, 25). Several HSP90 inhibitors have undergone human clinical trials against various cancer types (26, 27, 28, 29, 30, 31, 32, 33). Unfortunately, patients enrolled in clinical trials for cancer using compounds that inhibit HSP90 report several side effects, with night blindness being the most prevalent undesired outcome. Therefore, it is imperative to understand the function of HSP90 in the cell to design more effective HSP90-based medicine, avoiding side effects.

HSP90 comprises two independent but highly similar cytosolic paralogs: HSP90α, termed the stress-inducible, and HSP90β, the constitutively active paralog. Due to their sequence similarities, these paralogs are often not distinguished in studies. However, the animal models specific to each paralog have shown that these two paralogs have independent roles and may chaperone unique substrates in different cell types (34). The absence of HSP90β, albeit normal levels of HSP90α in a murine model, halted embryo development at Embryonic day 9.5 due to failed placenta labyrinth formation (35). The lack of HSP90α in the murine model causes infertility in the male reproductive system and vision loss despite the presence of HSP90β (36, 37). The client specificity of the cytosolic HSP90 paralogs, whether they have cell-type specificity, and what determines the division of labor between HSP90α and HSP90β is poorly understood.

Defects in PDE6 function and regulation are associated with retinal diseases and progressive photoreceptor degeneration (38, 39, 40). PDE6 is the effector enzyme in phototransduction signaling. In rod photoreceptor cells, PDE6 enzyme comprises a heteromeric catalytic core (α and β) and two inhibitory γ subunits. In comparison, cone photoreceptor cells express PDE6 with a homodimeric catalytic core (α') and two inhibitory γ' subunits (41, 42). The folding and assembly of the PDE6 are mediated by Aryl-hydrocarbon-interacting protein-like1 (AIPL1) (43), a known cochaperone of HSP90 (44). While the interaction of HSP90 and PDE6 is established, it is unknown whether both HSP90 cytosolic paralogs are needed to regulate PDE6 protein levels.

In this study, we developed a novel animal model lacking the HSP90α protein. HSP90α knockout model was designed to eliminate all the functional domains of HSP90α proteins with early premature termination. Our design avoids any possible secondary phenotype due to the accumulation of a non-functional fragment of the protein. The absence of HSP90α led to rod photoreceptor dysfunction at 2 months, with the accumulation of vacuolar structures and abnormalities in the outer segments. The decline in rod function was accompanied by progressive degeneration with complete loss of rods at 6 months.

Interestingly, the loss of HSP90α led to a reduction in rod phosphodiesterase 6 (PDE6). Our study shows the need for HSP90α in regulating the levels of rod PDE6 subunits; cone PDE6 was unaffected. In agreement with these findings, our studies show that rod function was affected while the cone function was preserved.

Our unbiased proteomics revealed potential novel HSP90α clients, including WDRs 17, 19, and 35, known as β-propeller domain proteins named after the number of Tryptophan-Aspartate (WD) motifs. Altogether, our work reveals a unique role for HSP90α in regulating the levels of rod PDE6 subunits and the AIPL1 cochaperone. In addition, this study demonstrates the requirement of HSP90α in rod and not cone photoreceptor maintenance.

## Results

### Design and confirmation of HSP90α knockout mouse

**Figure 1:**
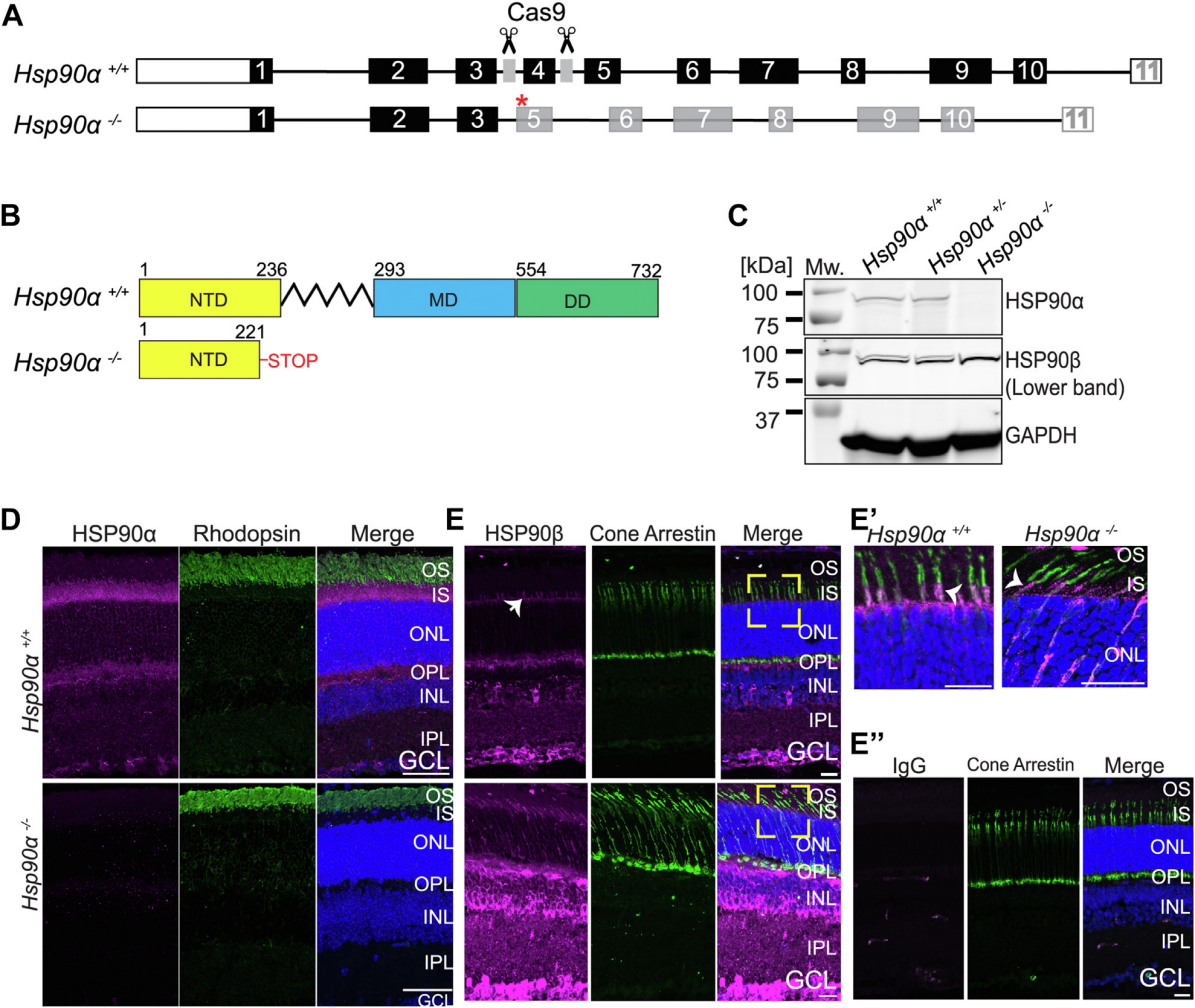
**Design and confirmation of HSP90α null mouse.***A*, schematic representation of *HSP90aa1* in the wild-type mouse (*Hsp90α*^*+/+*^) showing the location of the sgRNAs (*gray*) that target introns flanking exon 4 (*Top*). *Hsp90α*^*−/−*^ was generated after NHEJ following Cas9-mediated cleavage that led to exon 4 deletion and stop codon (*red asterisk*) in exon 5 (*Bottom*). *B*, organization of functional domains of HSP90α in *Hsp90α*^*+/+*^ includes NTD, MD, and DD. In *Hsp90α*^*−/−*^, a truncated fragment of NTD is predicted to be generated. The numbers on the top mark the number of amino acids at the start and end of each domain. *C*, immunoblotting of P45 retinal tissue lysate from *Hsp90α*^*+/+*^, *Hsp90*^*+/−*^, and *Hsp90α*^*−/−*^ mice using antibodies against indicated proteins. Standard protein molecular weight in kDa is indicated on the *left*. N = 3. *D*, retinal cross-sections from *Hsp90α*^*+/+*^ and *Hsp90α*^*−/−*^ animals at P30 stained with HSP90α (*magenta*), Rhodopsin (*green*), and DAPI (*blue*). *E*, retinal cross-sections from *Hsp90α*^*+/+*^ (*Top panel*) and *Hsp90α*^*−/−*^ animals (*Bottom panel*) at P30, stained with HSP90β (*magenta*), Cone arrestin (*green*), and DAPI (*blue*). *E’*, magnified image of the area in the *yellow bracket* from panel (*E*) showing the inner segment of photoreceptor cells. *Arrowhead* points to the HSP90β staining and colocalization with cone arrestin, a cone inner segment marker. *E”*, retinal cross-sections from *Hsp90α*^*+/+*^ animal stained with IgG antibody (*magenta*), cone arrestin (*green*), and DAPI (*blue*). Scale bar = 20 μm. DD, Dimerization Domain; GCL, retinal ganglion cell; INL, inner nuclear layer; IPL, inner plexiform layer; IS, inner segment; MD, Middle domain; NHEJ, non-homologous end-joining; NTD, N-Terminal Domain; ONL, outer nuclear layer; OPL, outer plexiform layer; OS, outer segment.

In this work, we assessed the role of the HSP90α chaperone in visual function using the HSP90α global knockout mouse model (*Hsp90α*^*−/−*^). We generated *Hsp90α*^*−/−*^ by CRISPR Cas9 by two guide RNAs that target the regions flanking exon 4 of the *Hsp90aa1* gene. The CRISPR-Cas9–mediated excision led to a deletion of 590 base pairs, removal of exon 4, and an early stop codon in exon 5 (Fig. 1*A*). The knockout allele is predicted to code for a truncated protein with a partial N-terminal domain fragment responsible for ATP binding, missing both the middle client binding domain and the C-terminal dimerization domain essential for HSP90 function (Fig. 1*B*). To confirm the loss of HSP90α, we performed immunoblotting using retinal tissues from *Hsp90α*^*−/−*^ and its littermate controls: *Hsp90α*^*+/−*^ and *Hsp90α*^*+/+*^. The retinal tissues from controls exhibited similar levels of HSP90α. On the other hand, HSP90α expression was ablated in the *Hsp90α*^*−/−*^ samples, while the expression of HSP90β was unaffected. GAPDH, the housekeeping protein, was used as a loading control (Fig. 1*C*).

In line with the phenotype observed in other studies using HSP90α murine models (37), HSP90α knockout males were infertile with no apparent sperm in the testis (Fig. S1) (37). To our surprise, the female knockouts were also infertile, suggesting that HSP90α may be essential in male and female reproductive systems (data not shown). Altogether, our findings demonstrated the successful generation of HSP90α knockout mice.

### HSP90α is mainly expressed in photoreceptor cells

To investigate the localization of HSP90, we used immunohistochemistry. Retinal cross-sections were stained with HSP90α antibody (magenta) along with an outer segment marker, Rhodopsin (green), and a nuclei marker DAPI (blue) (Fig. 1*D*). In retinal cross-sections from wild-type animals (*Hsp90α*^*+/+*^), HSP90α was mainly found in the photoreceptor inner segment, outer plexiform layer (OPL), and ganglion cell layer (GCL). The observed staining was specific since the signal from HSP90α was absent in retinal cross-sections from HSP90α knockout animals (*Hsp90α*^*−/−*^) (Fig. 1*D*).

Similarly, we stained the retinal sections from wildtype and HSP90α knockout animals with HSP90β antibody (magenta), cone arrestin (green), and DAPI (blue) (Fig. 1*E*). Retinal sections from *Hsp90α*^*−/−*^ and *Hsp90α*^*+/+*^ stained with HSP90 β antibody showed similar localization patterns. HSP90β colocalized with cone arrestin in the inner segment of the cone photoreceptors (Fig. 1*E’*-arrowhead). Additionally, HSP90β was found in the outer limiting membrane (Fig. 1*E*-arrow), OPL, and downstream retinal neurons, including the bipolar cell layer and ganglion cell layer (Fig. 1*E*). We noticed an increase in levels of HSP90β throughout the retina in *Hsp90α*^*−/−*^ animals. The upregulation of HSP90β was further confirmed in immunoblotting analysis (Figs. 1*C* and 4*B*). IgG-stained retinal sections served as a control and showed that the observed HSP90β staining was specific to the antibody used (Fig. 1*E”*).

To independently assess the expression pattern of HSP90 paralogs in retina layers, we compared the expression of HSP90 in retinal tissues where photoreceptors have completely degenerated (adult AIPL1 knockout) and a wild-type retina. HSP90α was highly reduced in retinal extracts lacking photoreceptor cells suggesting that photoreceptors express the vast majority of HSP90α found in the retina. In contrast, retinas without photoreceptors still expressed a significant amount of HSP90β, although a slight reduction was observed (Fig. S2). Overall, our data indicated that HSP90α was abundant in the inner segment of photoreceptor cells. In contrast, HSP90β expression was observed in the inner segment of cone photoreceptors and inner retinal neurons.

### The absence of HSP90α affects rod and not cone photoreceptor function

**Figure 2:**
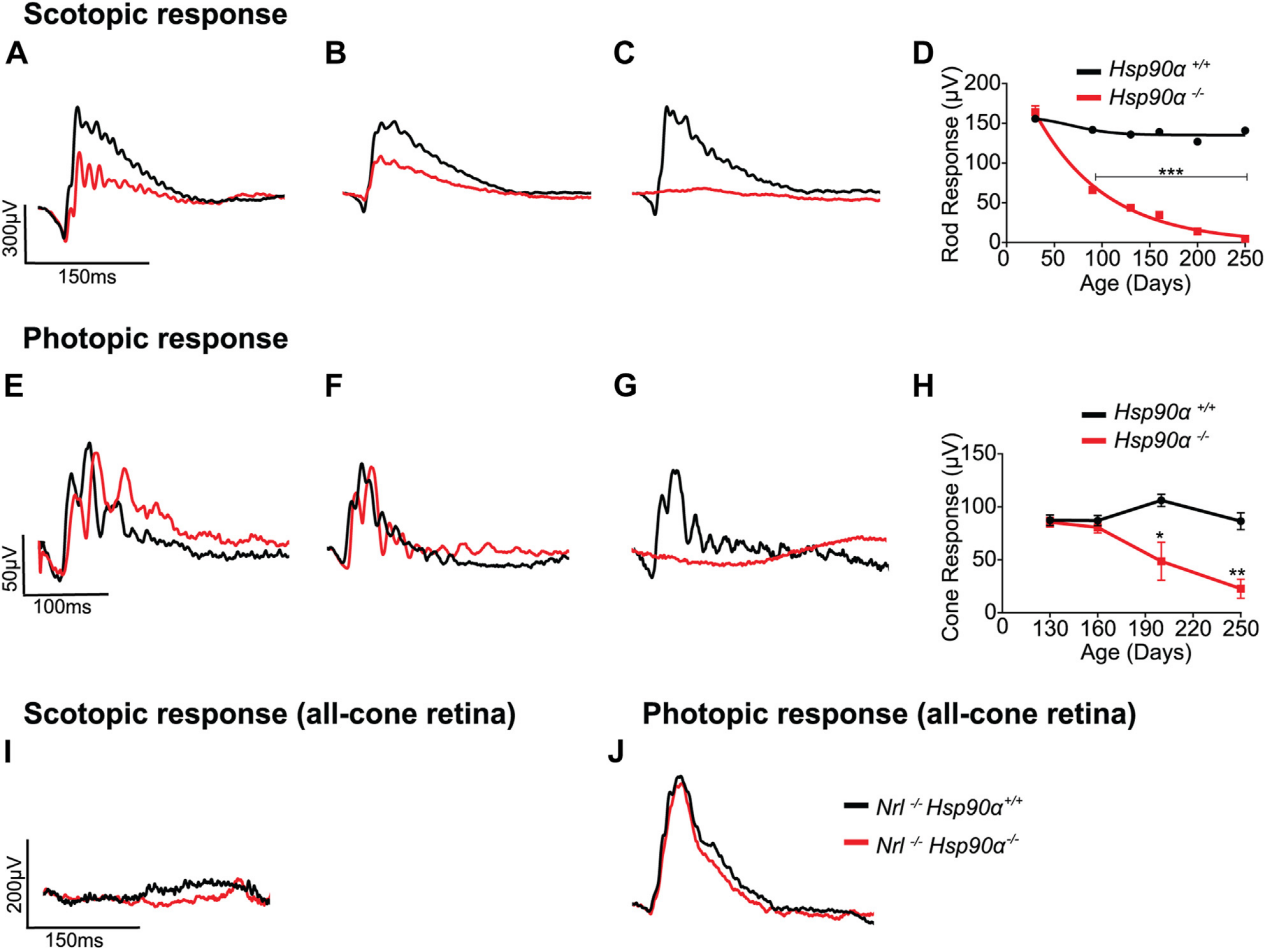
**Loss of photoreceptor function in the absence of HSP90α measured by ERG.***A*–*C*, representative traces of scotopic photoreceptor response from P30 (*A*), P160 (*B*), and P250 (*C*) animals measured at 0.025 cd s/m^2^ light intensity. Knockout: *Hsp90α*^*−/−*^ (*red*) and littermate control: *Hsp90α*^*+/+*^ (*black*). *D*, quantification of scotopic “a” wave response plotted against the age of the animal when the ERG was measured. *E*–*G*, representative traces of photopic response from P30 (*E*), P160 (*F*), and P250 (*G*) of *Hsp90α*^*−/−*^ (*red*) and *Hsp90α*^*+/+*^ (*black*) animals measured at 1.26 cd s/m^2^ light intensity. *H*, quantification of photopic “b” wave response plotted against the age of the animal when ERG was measured. *I* and *J*, photoreceptor response measured from *Nrl*^*−/−*^*Hsp90α*^*−/−*^ (*red*) and littermate control (*Nrl*^*−/−*^*Hsp90α*^*+/+*^) (*black*) recorded at P250. *I*, a representative trace of scotopic response at 0.025 cd s/m^2^ light intensity. *J*, photopic response recorded at 1.26 cd s/m^2^ light intensity. The recordings are of a minimum of four animals of both sexes. Statistical analysis was performed using two-way ANOVA with Sidak multiple comparisons t-tests using Graph Pad Prism with a *p*-value ≤0.05 (∗*p*≤ 0.05, ∗∗*p* ≤ 0.01, ∗∗∗*p* ≤ 0.001, and n.s = nonsignificant).

We assessed the visual function of *Hsp90α*^*−/−*^ and its littermate control, *Hsp90α*^*+/+*^, by electroretinography (ERG). This technique generates component waves originating from different retinal neurons. The “a” wave, trending downwards, originates from photoreceptor cells. The upward-trending “b” wave mainly represents the response from the bipolar cells (45). At postnatal day 30 (P30), both *Hsp90α* ^*−/−*^ and *Hsp90α* ^*+/+*^ had comparable scotopic (dark-adapted) “a” wave response indicating normal rod photoreceptor function (Figs. 2, *A* and *D* and S3*A*). The “b” wave response was reduced in *Hsp90α*^*−/−*^ (Figs. 2*A* and S3*D*), suggesting a defect in the coupling of photoreceptor cells to bipolar cells. *Hsp90α*^*−/−*^ mice showed a progressive reduction in both “a” and “b” wave response (Figs. 2, *B* and *D* and S3*B*) and complete loss of photoreceptor response at P250 (Figs. 2, *C* and *D* and S3*C*).

Despite the gradual loss of scotopic response, photopic (light-adapted) recording measuring cone photoreceptor function was unaffected in *Hsp90α*^*−/−*^ mice at P160 (Figs. 2, *E* and *F* and S3*E*). However, the photopic response was lost at P250 (Figs. 2, *G* and *H* and S3*F*), coinciding with a complete loss of rod function. In mouse models for retinitis pigmentosa, rod cell death triggers the loss of cones (46, 47, 48). Since the cone function was unaffected at P160, where we observed a significant reduction in rod response, we speculated that the decline in photopic response after P160 is likely due to an indirect effect caused by rod photoreceptor degeneration. To assess this hypothesis, we generated an all-cone NRL knockout mouse model (*Nrl*^*−/−*^) with or without HSP90α expression (Fig. S4*A*). The removal of the NRL transcription factor is known to generate an all-cone retina (49). Cone photoreceptors expressed both HSP90α and HSP90β paralog (Fig. S4*B*). HSP90β expression increased in *Nrl*^*−/−*^ *Hsp90α*^*−/−*^ similar to *Hsp90α*^*−/−*^ (Figs. 1*C* and S4*B*). As expected, the scotopic response of *Nrl*^*−/−*^ *Hsp90α*^*−/−*^ and *Nrl*^*−/−*^ *Hsp90α*^*+/+*^ littermate control was absent (Fig. 2*I*). The photopic response in *Nrl*^*−/−*^ *Hsp90α*^*−/−*^ mouse was indistinguishable from *Nrl*^*−/−*^ *Hsp90α*^*+/+*^ at P250 (Fig. 2*J*). These results showed that HSP90α is not needed for cone function and confirmed our hypothesis that in complete HSP90α knockout, cone degeneration was a bystander effect. Altogether, our finding shows that HSP90α is needed to maintain rod-mediated visual response.

### The progressive degeneration of photoreceptors in mice lacking HSP90α

**Figure 3:**
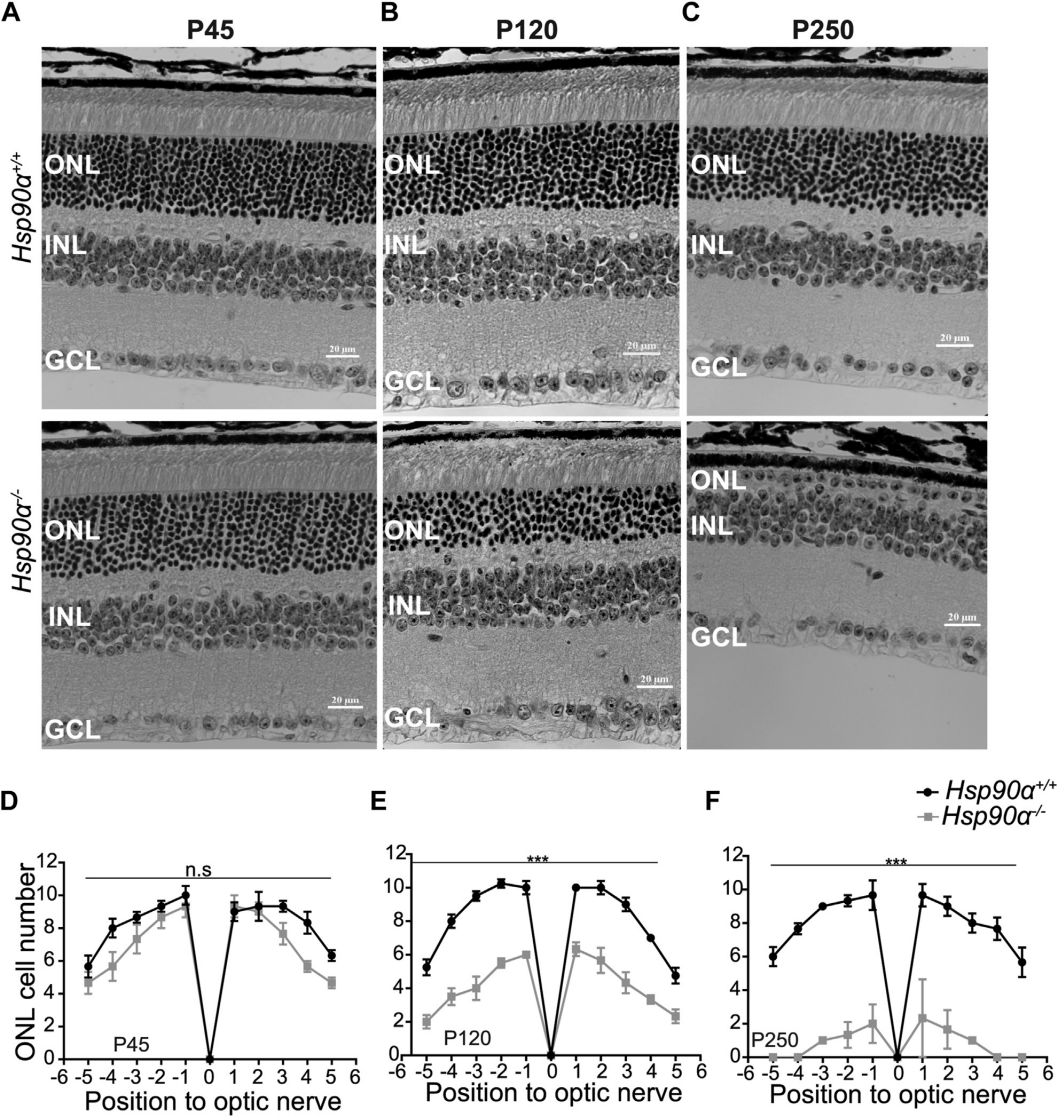
**Progressive photoreceptor degeneration in the retina lacking HSP90α.** Light microscopy of hematoxylin and eosin (H&E)-stained retinal cross-sections from *Hsp90α*^*+/+*^ (*Top*) and *Hsp90α*^*−/−*^ (*Bottom*) animals. ONL, INL, and GCL are shown at the indicated age. *A*, P45. *B*, P120. *C*, P250. *D*–*F*, spider plot depicting quantification of photoreceptor nuclei (ONL) in retinal cross-sections from *Hsp90α*^*+/+*^ (*black*) and *Hsp90α*^*−/−*^ (*gray*) mice. Photoreceptor nuclei from equally distanced regions on each side of the optic nerve (point 0) were counted at the indicated age. *D*, P45. *E*, P120. *F*, P250. Data are represented as mean ± SEM, N = 4, using multiple comparison t-tests using graph pad software (∗*p* ≤ 0.05, ∗∗*p* ≤ 0.01, ∗∗∗*p* ≤ 0.001 and n.s = non-significant). GCL, retinal ganglion cell; INL, inner nuclear layer; ONL, outer nuclear layer.

The vision loss observed in HSP90α knockout could be due to photoreceptor loss. Therefore, we stained retinal cross-sections of *Hsp90α*^*−/−*^ and littermate control with Hematoxylin and Eosin (H&E) to assess retinal morphology (Fig. 3). At P45, retinal layers appeared normal with no apparent changes in the number of photoreceptor nuclei (outer nuclear layer [ONL]) in HSP90α knockout compared to littermate control, suggesting normal retinal development in the absence of HSP90α (Fig. 3*A*). However, after P45, we observed progressive photoreceptor degeneration. At P120, the number of photoreceptor nuclei (ONL) in *Hsp90α*^*−/−*^ was reduced by half in the knockout, with 11 nuclei in the control *versus* five nuclei in the knockout (Fig. 3*B*). By P250, the HSP90α knockout retina had only one layer of photoreceptor nuclei left (Fig. 3*C*). Photoreceptor nuclei layers were uniformly and progressively reduced on each side of the optic nerve in the knockout retina after P45 (Fig. 3, *D*–*F*). The inner retinal layers were comparable between *Hsp90α*^*−/−*^ and control, even at P250. Although we did not detect noticeable photoreceptor loss until P60, TUNEL staining detected TUNEL-positive cells in the ONL of *Hsp90α*^*−/−*^ cross sections at P45 (Fig. S5*A*). In addition, we observed the activation and migration of microglia to the photoreceptor layers starting at P60 (Fig. S5*B*). From our findings, we infer that a decline in visual function is accompanied by microglia activation and progressive photoreceptor degeneration.

### HSP90α partially regulates levels of rod PDE6 protein

**Figure 4:**
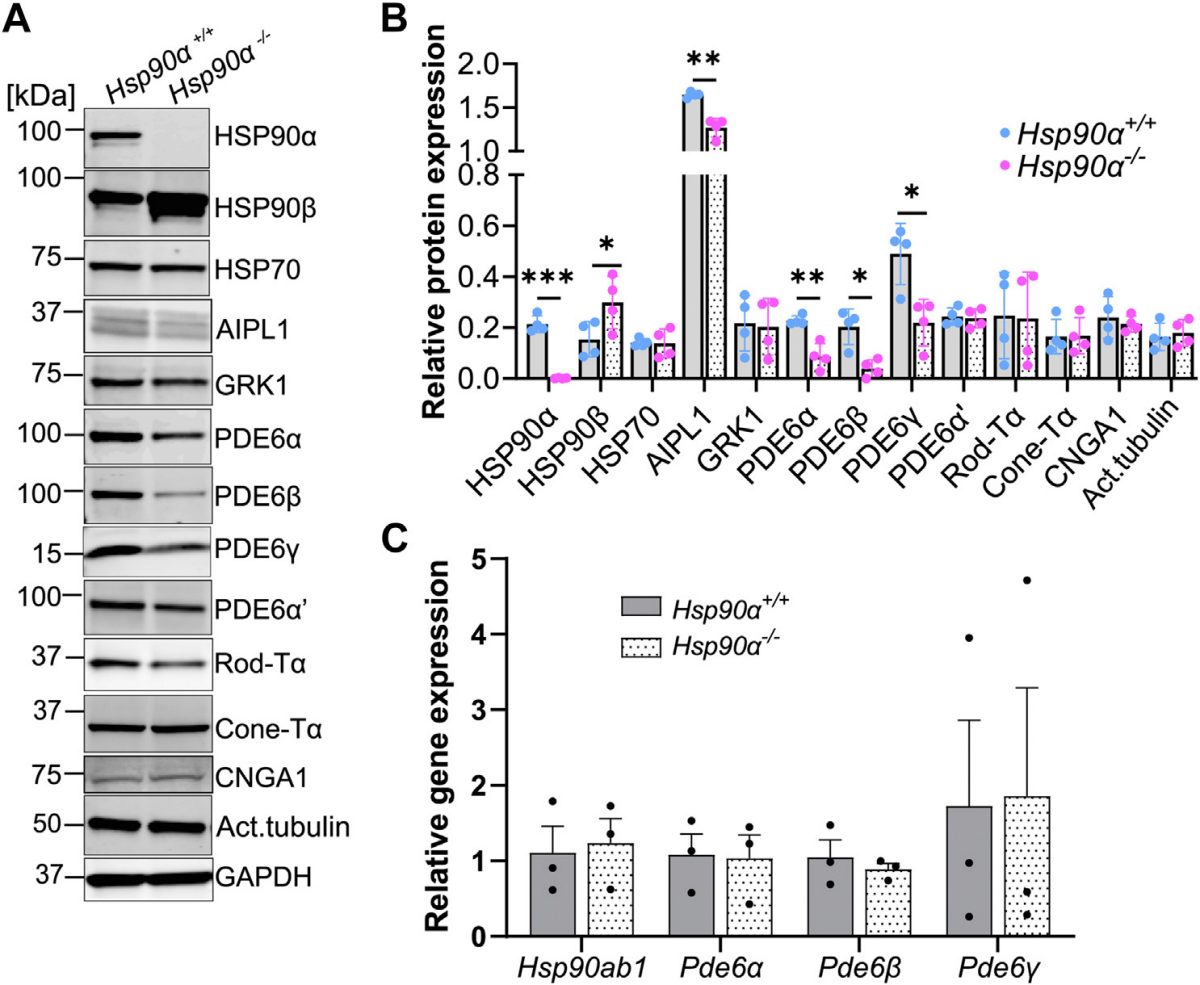
**Reduced levels of rod phosphodiesterase subunits in the HSP90α null retina.***A*, representative immunoblots of P15 retinal samples from *Hsp90α*^*+/+*^ and *Hsp90α*^*−/−*^ mice showing levels of indicated proteins. The molecular weight in KDa is indicated on the *left side* of the blot. *B*, densitometric analyses of immunoblots shown in (*A*) normalized to GAPDH. Relative protein expression is indicated in both *Hsp90α*^*+/+*^ retina (individual sample data shown in *blue*) and *Hsp90α*^*−/−*^ retina (individual sample data shown in *magenta*). Statistics are represented as mean ± SD of four replicates done in two independent experiments (∗*p* ≤ 0.05, ∗∗*p* ≤ 0.01, ∗∗∗*p* ≤ 0.001). *C*, relative mRNA levels of *Pde6* subunits and *Hsp90ab1*. Normalized transcript levels in retina extracted from *Hsp90α*^*−/−*^ relative to *Hsp90α*^*+/+*^. Statistics are represented as mean ± SEM with N = 3. All tested mRNA levels were not statistically significant.

We assessed protein levels through quantitative immunoblotting using retinal lysates from *Hsp90α*^*−/−*^ and littermate control. This method indirectly points to the potential substrates of HSP90α in photoreceptor cells (Fig. 4, *A* and *B*). We used retinal lysates obtained from tissue collected at P15 before the onset of degeneration. As expected, HSP90α was absent. We noticed that there was a twofold upregulation of HSP90β paralog. In contrast, the level of the HSP70 chaperone, an interactor of HSP90, was unchanged. Besides the chaperone proteins, we also checked the levels of phototransduction proteins. Previous studies suggested that HSP90 is vital in regulating G protein-coupled receptor kinases (GRKs), including the photoreceptor-specific kinase GRK1 (50, 51). In our study, GRK1 expression was unchanged in HSP90α knockout (Fig. 4, *A* and *B*). Murine retina treated with pan-HSP90 inhibitor caused a reduction in levels of the PDE6 enzyme (51). Since the compound used to inhibit HSP90 targets all the HSP90 paralogs, we set to assess if the HSP90α paralog is needed to maintain levels of PDE6. HSP90α knockout showed a 50% reduction of all rod photoreceptor PDE6 subunits (PDE6α, β, and γ) (Fig. 4, *A* and *B*). The cone-specific PDE6α' level was comparable to levels in control which align with our ERG finding that cone function is unaltered in the absence of HSP90α. Similarly, levels of PDE6α' were similar between knockout and control retina in an all-cone retina sample lacking HSP90α (Fig. S4*B*).

AIPL1, a cochaperone of HSP90, interacts with and regulates the stability and assembly of PDE6 (43, 44, 52). Since PDE6 expression was reduced, we assessed if AIPL1 levels were affected. To our surprise, AIPL1 levels were also reduced in HSP90α knockout. The decrease in AIPL1 expression was unexpected since HSP90 is not known to regulate the levels of most of its cochaperones. All other tested phototransduction proteins, including Rhodopsin, rod and cone-Transducin (Tα), and Cyclic Nucleotide Gated Channel Subunit α1 (CNGA1), were unaffected by the removal of HSP90α in the retina.

To test whether the change in protein levels occurs post-transcriptionally, we performed quantitative RT-PCR using mRNA extracted from the retinal lysate of *Hsp90α*^*−/−*^ and corresponding control at P15. We analyzed the message levels in *Pde6a*, *Pde6b*, *Pde6g*, and *Hsp90ab1* genes. The message levels were normalized to *Ywhaz*, a housekeeping gene, and represented as relative expression compared to the wild type (Fig. 4*C*). The expression of *Pde6* subunits and *Hsp90ab1* were unaffected by the removal of HSP90α (Fig. 4*C*). These findings imply that the changes in rod PDE6 and HSP90β protein expression occur post-transcriptionally.

Overall, the immunoblotting analysis showed that HSP90α plays a crucial role in rod signal phototransduction by regulating the levels of rod PDE6 and AIPL1 proteins. In addition, our results support the functional analysis by ERG (Fig. 2), which showed a loss of rod function but preserved cone function in animals lacking HSP90α. In line with this finding, rod PDE6 expression was affected, but cone PDE6 expression was unaltered in HSP90α knockout.

### Identification of differentially expressed proteins in the absence of HSP90α in the murine retina

**Figure 5:**
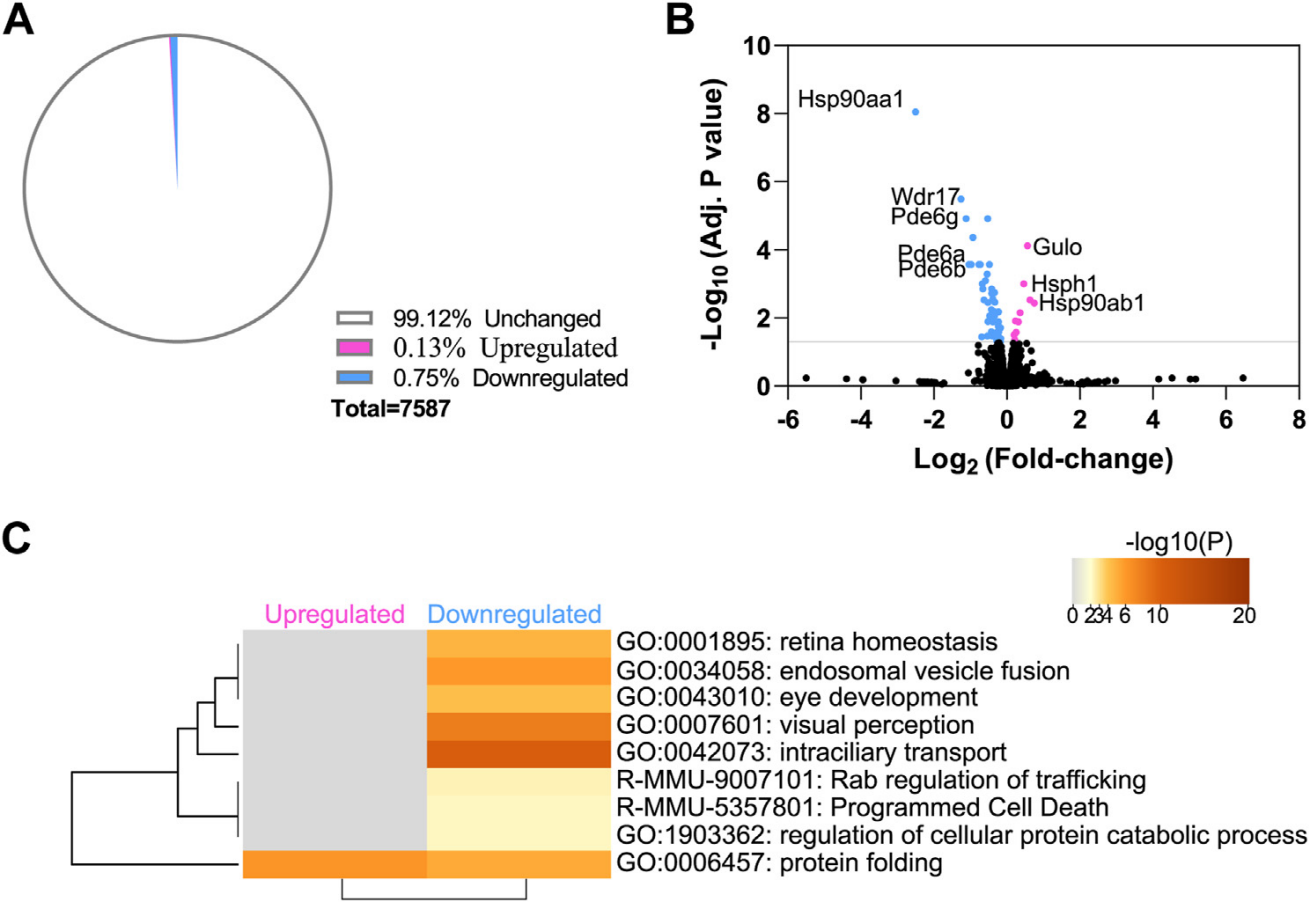
**Minimal proteome changes in the retina lacking HSP90α.** Quantitative proteomics analysis of retinal samples at P15 from *Hsp90α*^*−/−*^ and *Hsp90α*^*+/+*^mice. *A*, pie chart indicates the changes in levels of 7587 quantified proteins in *Hsp90α*^*−/−*^ retinas and their *Hsp90α*^*+/+*^ littermate controls. *White* (unchanged), *blue* (upregulated), and *magenta* (downregulated). *B*, volcano plot of statistical significance (−log_10_ (*p* value) against fold change (log_2_ (FC)) of all the quantified proteins. The top three upregulated and downregulated proteins are shown. *C*, heatmap showing the gene ontology enrichment analysis of upregulated and downregulated proteins in *Hsp90α*^*−/−*^ retinas. The statistical significance of the enrichment is indicated by color, from *gray* (low enrichment) to *dark orange* (high enrichment).

We conducted quantitative Tandem Mass Tags (TMT) proteomics to decipher the broad impact of HSP90α removal on retinal protein expression. Five retinas of wild type and knockout were collected at P15 when there was no defect in photoreceptor function or morphology (Figs. 2 and 3). Peptides extracted from the retinal tissues were labeled using plex-10 tags followed by LC-MS/MS analysis. TMT proteomics quantification provided the relative abundance of proteins in retinal samples in the presence or absence of HSP90α. A pie chart illustrates the number of quantified and differentially expressed proteins in the knockout (Fig. 5*A*). A total of 7587 proteins were quantified. Of all identified proteins, 67 were differentially expressed between the control and HSP90α knockout groups (Table S1). Fifty-seven proteins were downregulated, while 10 were upregulated in the knockout retina. A Volcano plot of adjusted *p*-value showing the statistical significance plotted against the fold change summarizes the proteome changes observed in the absence of HSP90α. Unchanged proteins are shown in black, upregulated ones in magenta, and downregulated proteins are indicated in blue (Fig. 5*B*). HSP90α was the most reduced protein considering all the detected peptides. As expected, there was no signal in the HSP90α knockout samples when HSP90α unique peptides were considered (data not shown). Besides HSP90α, the expression of tryptophan-aspartic acid (WD) repeat-containing protein 17 (WDR17) was significantly reduced. WDR17 is a protein with unknown function and is highly expressed in the retina and testis (53). Comparable to the quantitative immunoblotting (Fig. 4, *A* and *B*), rod-specific phosphodiesterase 6 subunits and AIPL1 proteins were reduced in the absence of HSP90α (Table S1). The upregulated proteins in the knockout retina included HSP90β paralog. Besides HSP90β, levels of HSP105, a member of the HSP70 family of proteins, were elevated. L-gulonolactone oxidase enzyme that catalyzes the last step in ascorbate (Vitamin C) synthesis (54) was among the most upregulated proteins in HSP90α knockout (Fig. 5*B* and Table S1).

To better understand the cellular pathways regulated by HSP90α, we used Metascape software to analyze the biological function and pathway enrichment of differentially expressed proteins (Fig. 5*C*). The protein folding group was the only upregulated pathway. It included heat shock proteins such as HSP90β, HSP105, and HSP40. Interestingly a smaller subset of folding proteins was downregulated. This group included mainly cochaperone proteins. Other downregulated proteins were linked to intraciliary transport, vesicle fusion, and proteins essential in retinal function and maintenance. In brief, findings from proteomic analysis aligned with those we found from quantitative immunoblotting. The proteomic analysis confirmed that the removal of HSP90α in the retina caused a reduction in rod PDE6 subunits, while cone PDE6 was unaffected. Proteomics analysis gave a broad spectrum of proteins that HSP90α regulates and potential clients unique to HSP90α paralog, including WD repeat-containing proteins. Removal of HSP90α affected a small set of proteins vital in retinal maintenance, emphasizing the need for HSP90α in several pathways critical in retina maintenance.

### The absence of HSP90α does not affect the morphology of photoreceptor cilia

**Figure 6:**
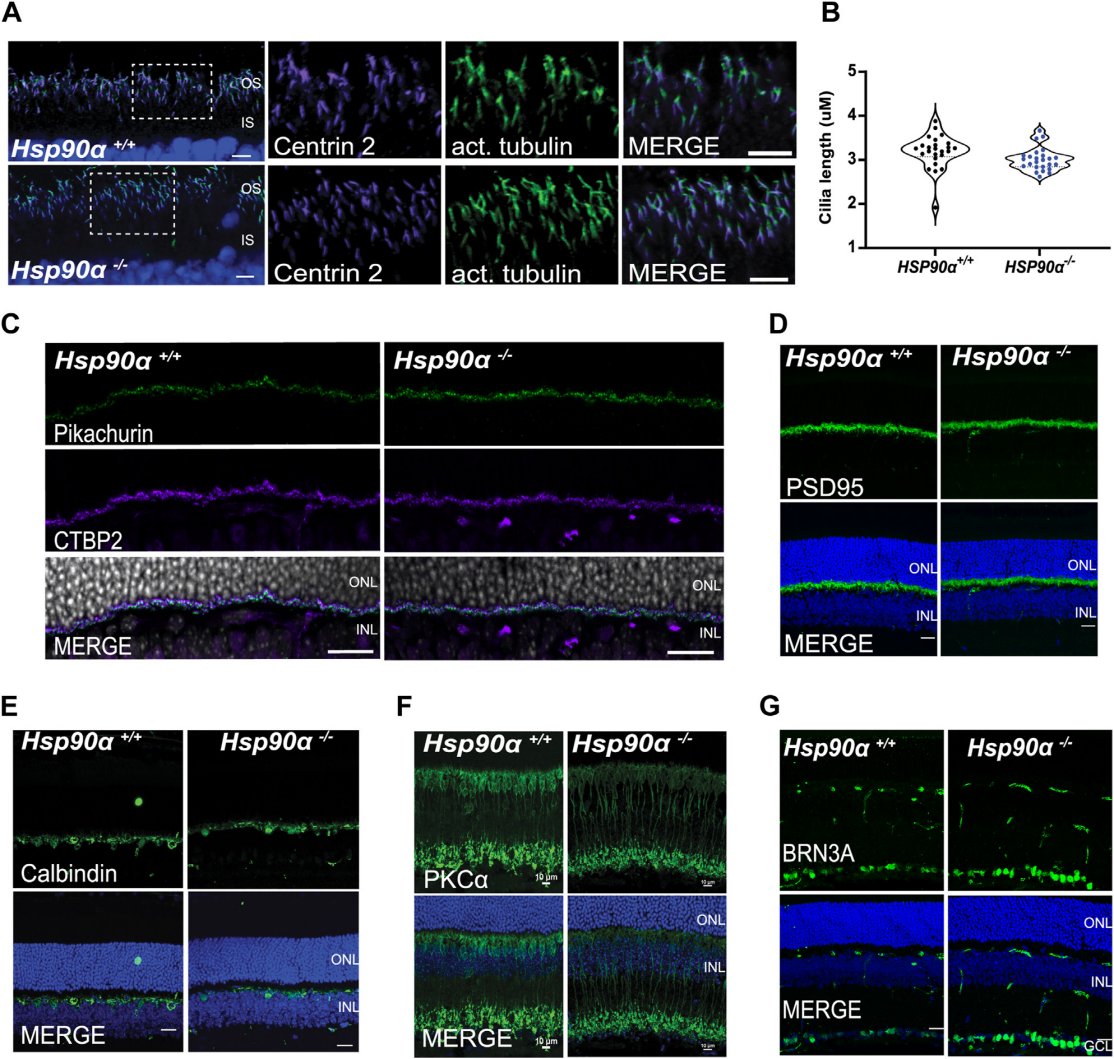
**Unaltered morphology of photoreceptor cilia and inner retinal neurons in the absence of HSP90α.** Representative retinal cross-sections from P30 *Hsp90α*^*−/−*^ mice and their littermate controls stained with (*A*) acetylated tubulin (*green*), DAPI (*blue*), and Centrin 2 (*magenta*) to assess the health of photoreceptor cilia. *B*, assessment of ciliary length by measuring the length of acetylated tubulin. Data are represented as mean ± SEM (N = 30, unpaired two-tailed *t* test; ∗*p* < 0.05). No significant change was observed in cilia length between *Hsp90α*^*−/−*^ and *Hsp90α*^*+/+*^ animals. *C*–*G*, retinal sections stained with synaptic and inner retinal neuronal markers. *C*, CTBP2 (magenta), DAPI (*gray*), Pikachurin (*green*). *D*, pre-synaptic marker PSD95 (*green*). *E*, horizontal cells stained with calbindin (green). *F*, bipolar cell marker PKCα. *G*, ganglion cells stained with an antibody against BRN3A (*green*). DAPI (*blue*) stains the nuclei. The scale bar is 20 μm.

Our proteomics data indicated differentially expressed proteins linked to ciliary transport. These proteins included: WD repeat-containing protein 35 (WDR35), retinitis pigmentosa GTPase regulator (RPGR), NudC domain containing 3 (Nudcd3), and Bardet-Biedl syndrome1 and 4 (BBS1 and BBS4, respectively) (Table S1). Additionally, studies have shown that HSP90 interacts with tubulin, an essential building block of the ciliary structure; inhibition of HSP90 altered cilia length in tissue culture cells (55, 56, 57). These findings prompted us to investigate the morphology of photoreceptor cilia in the absence of HSP90α. We used P30 retinal cross-sections from *Hsp90α*^*−/−*^ and littermate control to assess changes in the cilia before photoreceptor degeneration. We stained the retinal sections with acetylated tubulin (green) to mark the cilia and centrin 2 (magenta) to stain the connecting cilium. Both acetylated tubulin and centrin were properly localized (Fig. 6*A*). Cilia length measured using the acetylated tubulin staining was similar between the control and knockout samples (Fig. 6*B*). Our findings show that HSP90α does not play a critical role in photoreceptor ciliogenesis.

### Inner retinal neurons retain intact morphology in the absence of HSP90α

Functional analysis in HSP90α knockout showed a reduction in ERG “b” wave (Figs. 2*A* and S3*D*). Defects in synaptic transmission or defective synaptic morphology could be the root cause of the reduced ERG “b” wave. Furthermore, we observed HSP90α expression in the OPL connecting the photoreceptor with downstream neurons (Fig. 1*D*). The histological H&E analysis points to the normal development of inner retinal neurons and no signs of cell loss as indicated by the intact number of nuclei layers in INL and GCL in *Hsp90α*^*−/−*^ and control (Fig. 3, *A*–*C*).

We assessed the morphology of the inner retinal neurons using immunofluorescent microscopy. P30 retinal cross-sections were stained with known protein markers for different inner retinal neurons. C-terminal binding protein 2 (CTBP2), a photoreceptor ribbon marker, was co-stained with Pikachurin, an extracellular matrix-like protein found in the outer plexiform layer connecting the synapse of the photoreceptor, horizontal, and bipolar cells. CTBP2 showed a C-shaped staining pattern with punctate Pikachurin in the middle. The staining pattern was similar in both *Hsp90α*^*−/−*^ and control (Fig. 6*C*). We stained the retina with Postsynaptic density protein 95 (PSD-95) as an additional marker for the outer plexiform layer area. In the retina, PSD95 staining is found around the photoreceptor terminals (58). We did not observe any visible changes in PSD95 expression between *Hsp90α*^*−/−*^ and control (Fig. 6*D*). There was no noticeable difference in the structure of horizontal cells stained with Calbindin (Fig. 6*E*), bipolar cells stained with protein kinase C alpha (Fig. 6*F*), and retinal ganglion cell layer, marked by brain-specific homeobox/POU domain protein 3A (BRN3A) (Fig. 6*G*). Overall, there was no apparent defect in the morphology of inner retinal neurons before the onset of degeneration in HSP90α knockout. In addition, we did not find significant alterations in the levels of proteins expressed in the inner neurons. However, TMT proteomic analysis showed a reduction in proteins involved in vesicle-mediated transport and cellular catabolic pathways, which could affect the response transmission between retinal synapses. Further assessments are needed to pinpoint the pathways affected in HSP90α knockout.

### Photoreceptor structure in the absence of HSP90α

**Figure 7:**
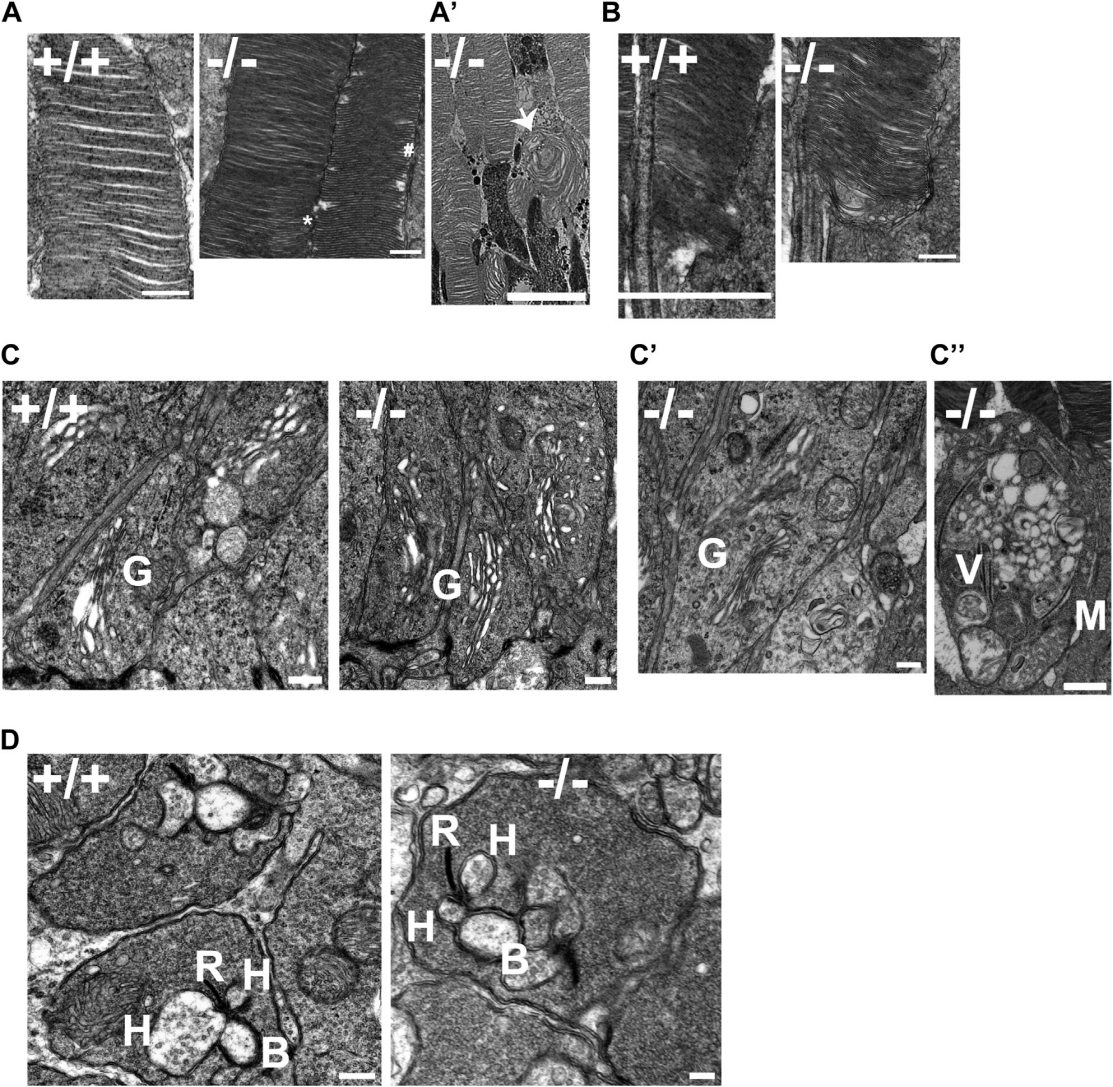
**Abnormal accumulation of vehicles in HSP90α knockout mice.** Ultrastructural analyses of photoreceptors by transmission electron microscopy at P30 and P60. *A*, normal structure and alignment of photoreceptor outer segment disks in *Hsp90α*^*−/−*^ (−/−) and littermate control, *Hsp90α*^*+/+*^ (+/+) at P30, scale bar 500 ոm. Rod outer segments discs (∗); and cone outer segment (#). *A’*, abnormal whorl-like structure (indicated by *arrow*) in *Hsp90α*^*−/−*^ outer segment at P60, scale bar 5 μm. *B*, photoreceptor connecting cilium in *Hsp90α*^*−/−*^ and *Hsp90α*^*+/+*^, scale bar 500ոm. *C*, normal Golgi stacks at P30, scale bar 500ոm. *C’*, Golgi (G) structure in *Hsp90α*^*−/−*^ animal at P60, scale bar 500ոm. *C”*, accumulation of structure filled with vesicles (V) in *Hsp90α*^*−/−*^ animal at P60, scale bar 1 μm. *D*, retinal synapse in the outer plexiform layer in *Hsp90α*^*−/−*^ and *Hsp90α*^*+/+*^ at P30, scale bar 500 ոm. TEM images shown here are a representation of three independent experiments. B, Bipolar cells synapse; H, Horizontal cell synapse; R, Ribbon structure

To explore the ultrastructural defects in *Hsp90α*^*−/−*^, we performed transmission electron microscopy (TEM) using *Hsp90α*^*−/−*^ retinal tissue and littermate control. At P30 before the onset of photoreceptor cell death, outer segment disks of rod photoreceptors were well aligned in the knockout, similar to wild-type control (Fig. 7*A*). Additionally, connecting cilia structure appeared normal in the knockout (Fig. 7*B*). TEM showed normal stacks of Golgi with no apparent abnormalities in the knockout at P30 (Fig. 7*C*). In addition, Golgi-mediated post-translational processing was unaffected as indicated by the normal glycosylation and trafficking of Rhodopsin in HSP90α knockout (Figs. S6*A* and S7*A*). Similarly, Golgi morphology and localization of GM130, a Golgi protein, appeared normal in the absence of HSP90α (Fig. S6*B*). Likewise, levels of other Golgi-specific proteins were unchanged in our proteomics analysis (Fig. S6*C*).

Since it was previously proposed that structural defect in Golgi was the cause of photoreceptor cell death in HSP90α knockout (36), we assessed whether the Golgi defect was delayed in our knockout model by checking the ultrastructure of Golgi at the initial stages of photoreceptor degeneration using P60 retinal tissue of *Hsp90α*^*−/−*^ and littermate control animals. Golgi stacks were indistinguishable between the knockout and control (Fig. 7*C’*). However, at P60, whorl-like structures were observed in the disc region (Fig. 7*A’*-arrow). We also observed an abnormal accumulation of vesicles in the inner segment (Fig. 7*C”*). Given the reduction in the “b” wave in ERG analysis at P30, we also checked the structure of the outer plexiform layer containing synapses of photoreceptor, horizontal, and bipolar cells. There were no noticeable structural defects in HSP90α knockout (Fig. 7*D*). Our retinal ultrastructural analysis showed normal maturation of photoreceptor outer segments followed by structural abnormalities in the OS disks.

### HSP90α interacts and regulates the levels of AIPL1 and rod PDE6

**Figure 8:**
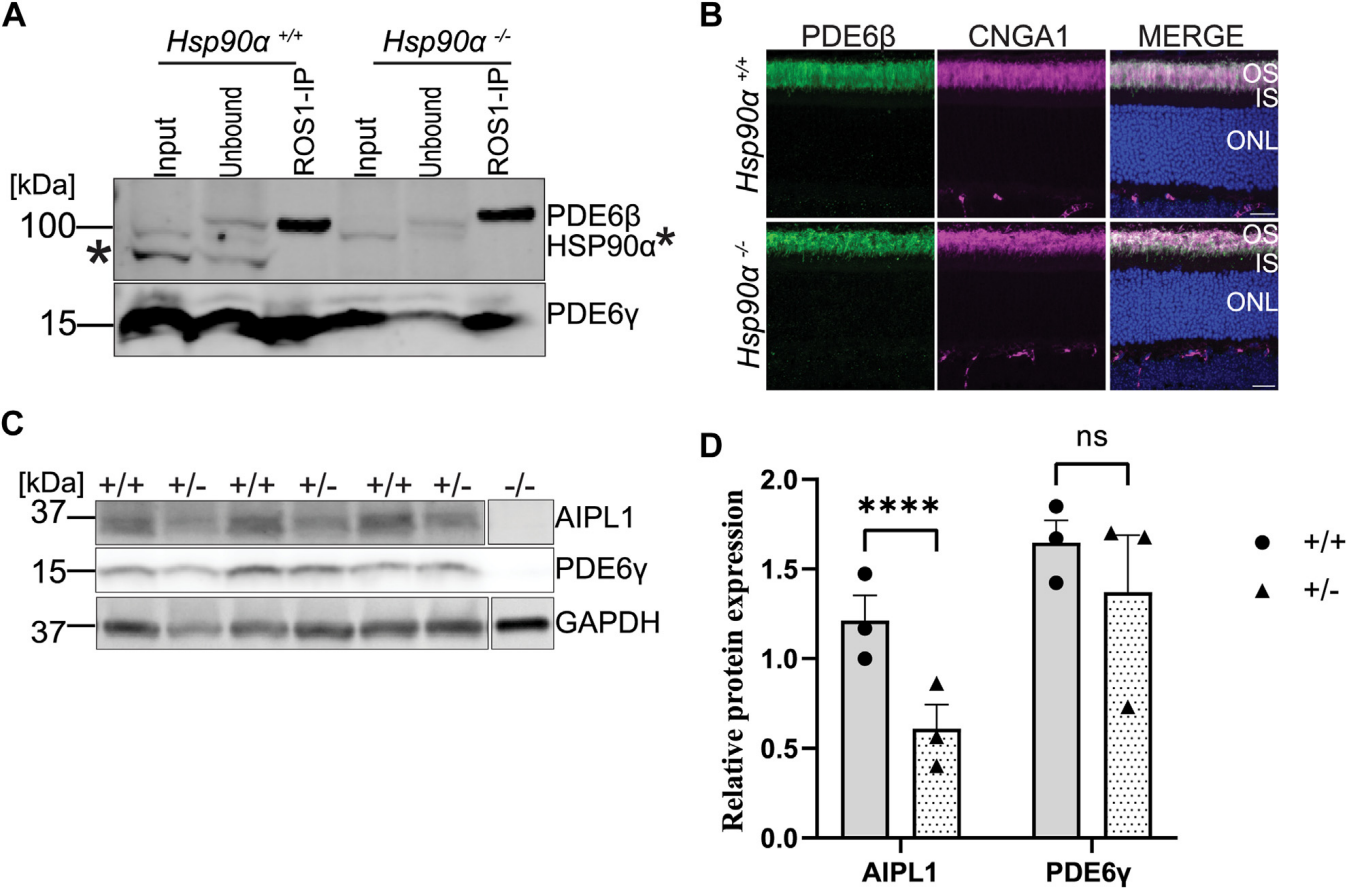
**HSP90α independently regulates the levels of AIPL1 and PDE6 proteins.***A*, assembly of PDE6 subunits in the absence of HSP90α, Representative image of three replicates. Immunoprecipitation of retinal extracts from P15 animals with ROS1 antibody (ROS1-IP) followed by immunoblotting with antibodies against PDE6β, PDE6γ, and HSP90α (band indicated with *asterisk*). Input = total protein in the retinal lysate, unbound = fraction not bound to the beads, and ROS1-IP = Eluted fraction. *B*, immunohistochemistry using retinal cross-sections obtained from P15 in *Hsp90α*^*+/+*^ and *Hsp90α*^*−/−*^ animals. Sections are stained with antibodies against PDE6β (*green*), CNGA1 (*magenta*), and DAPI (*blue*). Merge shows all the merged channels. The scale bar is 20 μm. *C*, immunoblots showing levels of indicated protein extracts from P80 retinal lysate from *Aipl1*^*+/+*^ and *Aipl1*^*+/−*^ animals. *D*, quantification of AIPL1 and PDE6γ proteins expression from immunoblots in panel (*C*). Normalized AIPL1 and PDE6γ protein levels against GAPDH. Statistics are represented as the mean ± SEM of three independent experiments (∗∗∗∗*p* ≤ 0.001, ns: non-significant). IS, inner segment; ONL, outer nuclear layer; OS, outer segment.

AIPL1 regulates the stability and assembly of the heterotrimeric PDE6 complex (43). Given the reduction of AIPL1 expression in HSP90α knockout, we tested whether the expressed PDE6 subunits assembled properly. We immunoprecipitated the PDE6 complex with ROS1 antibody, followed by immunoblotting with antibodies against individual PDE6 subunits. ROS1 is an established monoclonal antibody known to pulldown assembled and functional PDE6 complex (43). The eluted retinal lysate of both knockout and control showed all PDE6 subunits indicating that the residual PDE6 proteins in HSP90α knockout retain the ability to form a functional complex (Fig. 8*A*). In addition to assembly, we also assessed whether the absence of HSP90α impaired the translocation of PDE6 subunits to the outer segment compartment. Retinal cross-sections from *Hsp90α*^*−/−*^ and the control at P45 showed similar localization of PDE6β (Fig. 8*B*) and PDE6γ (Fig. S7*A*) to the outer segment. Additionally, all the other phototransduction proteins, including Rhodopsin, CNGA1, GRK1, peripherin, and transducin α, were found in their expected location (Fig. S7, *B* and *C*). HSP90 is a known interacting partner of AIPL1, a cochaperone for PDE6 (59). Immunoprecipitation of AIPL1 from bovine retinas followed by MS indicated that HSP90α paralog and rod PDE6 were the interactors of AIPL1 (data not shown). In this study, our proteomics results indicated that HSP90α paralog regulates the levels of AIPL1 (Table S1). Given the contribution of HSP90α maintaining levels of AIPL1 and PDE6, it raised the question of whether the photoreceptor defect observed in *Hsp90α*^*−/−*^ was due to reduced levels of AIPL1. To obtain better insight into the interaction among these three proteins, we assessed PDE6 levels in the retina obtained from AIPL1 heterozygous mice (Fig. 8, *C* and *D*). As expected, the retina with one functional copy of AIPL1 showed a 50% reduction in AIPL1 levels, similar to what we observed in this study in *Hsp90α*^*−/−*^ animals (Fig. 4, *A* and *B*). Unlike HSP90α knockout animals, the retina expressing 50% of AIPL1 protein did not show any changes in PDE6 levels (Fig. 8, *C* and *D*).

## Discussion

Here, we analyzed the role of HSP90α paralog in visual function using a novel mouse model lacking HSP90α. We designed our murine knockout model to delete all functional domains of HSP90α. HSP90α knockout males were infertile and unable to produce sperm, agreeing with previous findings (37, 60, 61). The photoreceptor developed normally but displayed progressive degeneration that was complete at 6 months. Interestingly, the deletion of HSP90α led to a significant reduction in rod PDE6, an enzyme crucial for vision. In the sections below, we discuss the likely mechanisms for the need for HSP90α in rod photoreceptor cells.

We found that HSP90 cytosolic paralogs have a distinct spatial expression in the retina apart from the established changes in temporal expression. The expression of HSP90 paralogs changes as the retina matures. While both paralogs are expressed in the developing retina, HSP90β expression is reduced in the adult retina, implying that HSP90β may be essential in retinal development (36). In contrast, the expression level of HSP90α in the retina is maintained in adult animals indicating that HSP90α may play a role in retinal maintenance (36). Our study found that HSP90α expression was high in photoreceptor cells while HSP90β was abundant in the inner retina. HSP90α was expressed in the inner segment of photoreceptor cells, OPL, and GCL. In contrast, HSP90β was expressed in the inner segments of cone photoreceptor cells, the outer limiting membrane, and the inner retina.

We demonstrated that HSP90α was vital for rod function (Fig. 2). However, HSP90α was dispensable in cones. We confirmed the expendability of HSP90α in cones in a mouse model with an all-cone retina. Our results suggest that HSP90β is expressed in cones and that HSP90β is important in cone cell function.

The photoreceptor development and function were normal at P45, showing that HSP90α does not play a role in the early development or maturation of photoreceptor cells. After this P45, photoreceptors degenerated, and a vast majority of photoreceptors were lost at P250 in the absence of HSP90α. One interesting change we observed is the reduction in the levels of AIPL1 cochaperone (Fig. 4, *A* and *B*). We and others have proposed that AIPL1 interacts with HSP90 to regulate the assembly and stability of the PDE6 subunits (43, 52, 62, 63, 64). In line with previous data showing that pan-HSP90 inhibitors caused a reduction in PDE6 and GRK1 levels (51), the expression of rod PDE6 was significantly reduced in the knockout mice. All three subunits of rod PDE6 but not cone PDE6 were reduced. These findings were confirmed by immunoblotting and proteomic analysis. HSP90α regulates PDE6 levels post-transcriptionally since the message levels were unaltered. Furthermore, HSP90α is not essential in the assembly of PDE6 subunits.

Mutations affecting the expression of PDE6 and AIPL1 cause retinitis pigmentosa and Leber congenital amaurosis with associated photoreceptor degeneration (39, 40, 43, 65). Nevertheless, the HSP90α knockout retina expressed more than 50% AIPL1 and functional PDE6 since photoreceptor function was normal until P45, despite changes in PDE6 levels. In an AIPL1 hypomorph with about 20% to 25% AIPL1 expression, a significant reduction (20% of wildtype) in rod PDE6 subunits was observed (63). Additionally, photoreceptor function and morphology were preserved until the retina was fully developed, followed by progressive degeneration and loss of photoreceptors in the AIPL1 hypomorph. Altogether, there is a precedence that altered levels of AIPL1 lead to a reduction in PDE6 and photoreceptor degeneration. In AIPL1 heterozygous animals, where AIPL1 was reduced by 50%, similar to our observation in the HSP90α knockout model, we do not find a significant change in PDE6 levels (Fig. 7, *C* and *D*). Our findings suggest that the reduction in PDE6 levels in the absence of HSP90α and subsequent degeneration is unlikely to be primarily driven solely by changes in AIPL1. The levels of GRK1, a kinase regulated by HSP90, were not affected, suggesting that HSP90β may regulate GRK1 levels in the absence of HSP90α.

To uncover the broader impact of HSP90α on the retinal proteome, we performed unbiased proteomics comparing the protein levels in the retina from wild-type and knockout animals. We used retinal tissue from P15 animals well before photoreceptor degeneration. Interestingly, the absence of HSP90α affected the expression levels of less than 1% of 8000 quantified retinal proteins (Fig. 4*A*), demonstrating the specificity of the proteomic analyses and the role of this HSP90α in the maintenance of a subset of retinal proteins. Molecular chaperones, HSP90β paralog, HSP105, HSP70 interacting protein (ST13), and HSP40 were upregulated (Table S1). HSP90β levels were unchanged in the retina of previously established models of photoreceptor degeneration, including ARL2BP and AIPL1 (64, 66) (Fig. S8). The latter results indicated that the upregulation of HSP90β paralog is uniquely linked to the absence of HSP90α.

On the other hand, there was a reduction in different cochaperones, including NudC domain-containing protein 3 (Nudcd3), cell division cycle 37, and tetratricopeptide domain-containing cochaperone that interacted with MEEVD sequence on HSP90's C-terminal domain. These cochaperones included immunophilin family FKBP51, FKBP38, Tetratricopeptide repeat protein 21B, AIP, and its homolog AIPL1. These changes were surprising since the expression of the cochaperone was thought to be independent of their interacting chaperone (67). The effect of HSP90α removal on the levels of cochaperones suggests possible interactions and unique feedback loops in the chaperone system.

The absence of HSP90α led to the reduction of two E3 ligase-related protein expression levels. These proteins are Kelch-like protein 18, a member of the Kelch-like gene family which interact with Cullin 3 to form an E3 ligase complex (68), and HECT Domain Containing E3 Ubiquitin Protein Ligase 3 (Herc3) that facilitates ubiquitin-mediated degradation by the proteasome (69). We reasoned that the reduction in the above E3 ligase proteins and the upregulation of HSP90β could contribute to the proteasome system malfunction. We used a model with a constitutively accessible ubiquitin substrate to assess the ability of proteasome to clear the substrate (70). In the absence of HSP90α, the proteasome prevented the accumulation of the constitutively active ubiquitin (Fig. S9). The latter results suggest that HSP90α knockout did not cause a significant overload and impairment of the proteasome system.

Exposure to light is known to cause photoreceptor cell death (71, 72, 73). HSP90α paralog is known to be the stress-inducible paralog, while HSP90β is constitutively active in the cell. We reasoned that the absence of HSP90α accelerated light-stress-mediated degeneration. However, HSP90α knockout underwent a similar rate of degeneration and functional loss when raised in the complete dark *versus* the normal vivarium light-dark conditions (data not shown). This finding rules out light exposure as a trigger for photoreceptor degeneration observed in HSP90α knockout.

A previous study on HSP90α suggested defective cilia as a cause of photoreceptor degeneration (36). Although the functional enrichment of differentially expressed proteins in our proteomic analysis contained ciliary proteins, the cilia morphology was normal in the knockout. HSP90α was proposed to interact with microtubule-associated protein (MAP1B). Reduction in MAP1B is linked to defective tubulin acetylation and was proposed to be the primary driver of photoreceptor degeneration in the absence of HSP90α (36). We did not notice a change in the expression and localization of acetylated tubulin in the absence of HSP90α (Figs. 4, *A* and *B* and 6, *A* and *B*). In addition, MAP1B expression was unchanged in the quantitative retinal proteome of wildtype and HSP90α knockout mice (Fig. S6*C*). Our work on α-tubulin acetyltransferase 1 knockout animal that lacked tubulin acetylation in photoreceptors showed no cell or functional loss even in the older animals refuting the hypothesis that defective tubulin acetylation drives photoreceptor degeneration (data not shown).

Additionally, the same study demonstrated that structural defects in Golgi as one of the causes of photoreceptor cell death in HSP90α knockout (36). However, we did not observe changes in Golgi or a reduction in Golgi-specific proteins (Fig. S6*C*). We speculate that the differences in phenotype observed are linked to the differences in the animal model used. By deleting all the functional HSP90α domains, we excluded phenotypes that may be related to the production of a large non-functional fragment of HSP90α leading to cellular stress (36). It is unlikely that the variability in mouse strain backgrounds caused the phenotype differences between studies since both studies used C57Black6/J mice.

WDR17, a β-propeller protein of unknown function, was significantly reduced in the HSP90α knockout retina. WDR17 is a novel gene highly expressed in the retina and testis (53). WDR17 is found in a cluster of genes linked to synaptic function. The reduced WDR17 may lead to the reduced “b” wave we observed in HSP90α knockout at an early age. Additionally, WDR17 was found among genes correlating with rhodopsin expression and mapped to human loci associated with retinitis pigmentosa (53, 74, 75). More studies will be needed to understand the function of WDR17 in the retina. Other WDRs, such as WDR19 and WDR35, were also reduced. A study characterizing the interaction network of chaperone-cochaperone-clients using mass spectrometry coupled to quantitative LUMIER assays uncovered that NUDC family cochaperones were associated with β-propeller folds, which includes WDRs and Kelch proteins (76). The reduction of WDR17, WDR19, WDR35, Kelch-like protein 18, and NUDCD3 expression suggests the disruption of the substrate-cochaperone complex in the absence of HSP90α.

In recent years, genetic studies using animal models have uncovered the role of HSP90α in different cells. Our study adds to an ongoing effort to understand the unique role of HSP90α in retinal tissue and the possible clients regulated by HSP90α. Our analysis of HSP90 in vision suggests overlap and possible redundancy in the function of HSP90α and its paralog HSP90β. We observed a 50% reduction in rod PDE6 and no change in cone PDE6 levels suggesting a unique role for HSP90β in cones. In addition, the upregulation of the HSP90β in the absence of HSP90α points to possible compensation, albeit poorly in slowing the photoreceptor degeneration. In the future, genetic studies using a double knockout of both paralogs are needed to assess overlap and unique clients of each HSP90 paralog role in the retina and other tissues. So far, genetic studies using knockout showed two main phenotypes, including sterility in males and vision loss. Our preliminary observation indicates that female knockouts were sterile. While our study points to a reduction in PDE6 as the cause of rod photoreceptor degeneration, we cannot exclude the possibility that defects in multiple pathways contribute to vision loss. For example, changes in WDR17, a gene of unknown function highly expressed in the retina with links to RP, could contribute to degeneration in conjunction with a reduction in PDE6 levels. Future studies are underway to uncover the connection between photoreceptor viability and loss of HSP90α in the retina.

The present study illustrates that HSP90α based drugs may not be practical due to visual impairment, especially if prolonged exposure to the inhibitor is needed. Understanding the role of HSP90 in the cell provides the essential knowledge required to design more reliable drugs and improve patient outcomes.

## Experimental procedures

### Design and genotyping of animal models

*HSP90aa1* knockout mouse was generated using CRISPR/Cas9 technology at mouse transgenic and gene targeting core (Emory University, Atlanta, Georgia). We used the http://crispr.mit.edu/ site to identify guide RNAs with the best cutting efficiency and lowest off-site activity. Synthetic single-stranded guide RNAs were acquired as CRISPR evolution sgRNA EZ Kit from Synthego. Two guide RNAs (5′-ccacaatcctcttcagataccac-3′ end and 5′-cctgaagctccctttagattaa-3′) in intron regions flanking exon 4 of *HSP90aa1* (ENSMUSE00001252094) were designed and injected with Cas9 nuclease in the blastocyst. The heterozygous founders that lacked exon 4 were identified by sequencing. The founders were crossed and maintained in the C57black6/J background. Genotyping was performed using genomic DNA extracted from ear punch samples. A multiplex PCR using two sets of PCR primers was used for genotyping. Set 1 includes Forward-A: 5′-CAATTGTAGGGGGTGTCTGG-3′ and reverse-A: 5′-TCCTCCTCTTCTTCATCAGAGC-3′ flanking the targeted region and amplifying a product of 820 and 336 base pairs from the wildtype and knockout samples respectively. Set 2 primers to amplify the wildtype DNA and consist of Forward-B: 5′-TTTGTGGGGAAGGTTAGCTG-3′ and reverse-B: 5′-TGGGATAGCCAATGAACTGA-3′.

Nrl knockout (*Nrl*^*−/−*^) was a generous gift from Dr Anand Swaroop at National Eye Institute (also at JAX labs, Strain #:021152). We crossed *Nrl*^*−/−*^ with heterozygous *Hsp90α*^*+/−*^ to generate an all-cone mouse lacking HSP90α. The genotyping for the *Nrl* allele was performed using *Nrl*-WT primers (5′-GTGTTCCTTGGCTGGAAAGA-3′ and 5′-CTGTTCACTGTGGGCTTTCA) and *Nrl*-KO primers (5′TTTCTGGTTCTGACAGTGACTACG-3′ and 5′-ACCAAATTAAGGGCCAGCTTCCT -3′).

Double knockout of *HSP90α* and Ub^G76V^-GFP transgenic mice was generated by crossing a GFP-expressing mouse with a heterozygous *HSP90α* mouse. Ub^G76V^-GFP transgenic mouse line was a generous gift from Dr Maxim Sokolov at West Virginia University and originated from Jackson Labs (B6.Cg-Tg (CAG-Ub∗G76V/GFP) 2 Dant/J Strain #:008112). Ub^G76V^-GFP transgenic mouse expresses a constitutively active ubiquitin with green fluorescent protein (GFP) reporter. Ub^G76V^-GFP animal is used to assess the status of the proteasomal system. The GFP reporter was genotyped using the following PCR primers (5′CCTACAGCTCCTGGGCAACGT-3′ and reverse: 5′-TCGACCCTTCCCCACCAC-3′.

The animals were raised in 12-h light/12-h dark cycles with food and water provided *ad libitum*. All experimental procedures involving animals in this study were approved by the Institutional Animal Care and Use Committee at West Virginia University.

### Immunoblotting

Mice were euthanized by CO_2_ inhalation followed by cervical dislocation. Eyes were enucleated, and retinas were dissected and flash-frozen immediately. Retinas were homogenized by sonication in lysis buffer (0.1% Triton X-100, 50 mM Tris with pH 7.5, 300 mM NaCl, and 5 mM EDTA, with protease inhibitor mixture (Roche)). Cellular debris was cleared at 4 °C by centrifugation for 30 s at 12,000*g*. Protein concentration was measured by a BCA assay kit (Thermo-Fisher Scientific). Samples were reduced by adding 1× SDS sample buffer (2% SDS, 10% Glycerol, 5% 2-Mercaptoethanol, 0.002% Bromophenol blue, and 62.5 mM Tris, pH 6.8) and boiled for 5 min before SDS-PAGE analysis. 70 μg of proteins from each sample were loaded in an individual lane and separated by SDS-PAGE gel. Next, we transferred the proteins onto the polyvinylidene difluoride membrane (Immobilon-FL; Millipore). After transfer, we proceeded with total protein stain (LI-COR Biosciences) used for protein normalization and quantification. The membrane was incubated in a blocking buffer (Odyssey Blocking Buffer; LI-COR Biosciences) for 30 min at room temperature. After incubation, the membrane was stained with the indicated primary antibodies and incubated overnight at 4 °C on a bidirectional rotator (Table S2). Antibodies were diluted into a 1:1 ratio of blocking buffer (Rockland) and PBS-T (Phosphate-buffered saline (PBS)/0.1% Tween-20). Following primary antibody incubation, the membrane was washed in PBS-T three times for 5 min each at room temperature and incubated with secondary antibodies (1:50,000 dilution in PBS-T), namely: goat anti-rabbit Alexa Fluor 680 (or 800) and goat anti-mouse Alexa Fluor 680 (Invitrogen) for 30 min at room temperature. After three washes of 5 min each with PBS-T, we imaged the membrane using Odyssey Infrared Imaging System (LI-COR Biosciences). Proteins detected by immunoblotting were normalized to the total protein stain and GAPDH. Statistical analysis and graphs were generated using GraphPad Prism software.

### Immunoprecipitation

PDE6 assembly in the retinal tissue of HSP90α knockout animals was assessed using immunoprecipitation. Retina from control and *Hsp90α* ^*−/−*^ were sonicated in 200 μl of lysis buffer (50 mM Tris, pH 7.4, 300 mM NaCl, 5 mM EDTA, and 0.02% Sodium Azide) containing protease inhibitors, 10 mM of iodoacetamide and 1% Triton X-100. The lysate was centrifuged at 14,000*g* for 30 s, and the supernatant was incubated with antibodies (AIPL1, PDE6, or ROS-1) for 2 h at 4 °C. To couple antigen-antibody complex to the beads, the lysate mixture was further incubated with protein A/G agarose beads for 3 h at 4 °C. We removed the unbound proteins (Unbound) by centrifugation at 14,000*g* for 30 s at 4 °C. We washed the beads three times with 1 ml of the wash buffer (0.1% Triton X-100, 50 mM Tris pH 7.4, 300 mM NaCl, 5 mM EDTA, and 0.02% Sodium Azide). After a final wash with 1 ml of 1× PBS, we eluted the sample in 1× SDS sample buffer. The samples were boiled for 5 min, then separated on SDS-PAGE, followed by immunoblotting using AIPL1 and PDE6 antibodies. The blot was imaged using Odyssey Infrared Imaging System (LI-COR Biosciences).

### Immunohistochemistry and histology

Eyes were enucleated and submerged in 4% paraformaldehyde (PFA) fixative (16% PFA solution EM grade diluted with PBS (Electron microscopy sciences)). After 15 min in the fixative, the cornea was removed, and the eyes were further fixed for 1 h and 45 min. The eyes were then washed in 1× PBS three times for 5 min each and incubated in an increasing concentration of sucrose diluted in PBS (7.5%, 15%, and 22% sucrose) for a minimum of 2 h on a nutator at 4 °C. Following sucrose incubation, eyecups were placed in a 1:1 ratio mixture of 22% sucrose and Cryo Optimal Cutting Temperature (OCT) Compound (Sakura) for 1 h. The lens was removed, and the eyecup was flash-frozen in OCT. The frozen eyecups were sectioned to 16 μm thickness using the Leica CM1850 cryostat, mounted on Superfrost Plus slides (Thermo Fisher Scientific), and stored at −20 °C. The sections were washed with PBS and incubated for 1 h in blocking buffer (PBS with 5% Goat sera, 0.5% Triton X-100, and 0.05% Sodium Azide) at room temperature and then incubated with primary antibody overnight at 4 °C. After removing the primary antibody (Table S2), sections were washed three times for 10 min in PBS-T (PBS and 0.5% Triton X-100). The sections were then incubated with secondary antibody incubation for 2 h at room temperature. Sections were washed three times each for 10 min in PBS-T and after the addition of ProLong Gold antifade (Thermo Fisher Scientific), the sections were coverslipped (Thermo Fisher Scientific). The stained retinal cross-sections were imaged using Nikon C2 Microscope. We used the FIJI-ImageJ software (National Institutes of Health) for additional image processing. Histological analysis was done using H&E staining at Excalibur Pathology.

### Electroretinogram

We performed ERG using the Celeris system (Diagnosys) with fully integrated electrodes built into the stimulator. After an overnight dark adaptation, mice were anesthetized using 1.5% isoflurane mixed with oxygen at a flow rate of 2 l/min. Body temperature was maintained at 37 °C with a regulated heating platform. The pupils were dilated using a 1:1 ratio solution of 8% tropicamide and 1.5% phenylephrine hydrochloride. Hydroxypropyl methylcellulose (Novartis Pharmaceuticals) was added to the cornea to protect and facilitate electrode contact. The reference electrode was placed between the ears and the ground electrode on the thigh of the left leg. The response from each eye was measured simultaneously from the corneal surface. Scotopic responses were obtained in complete darkness using white light flashes varying from 2.45 × 10^−4^ to 2.4 cd per second over a meter square. Following the scotopic recording, mice were light adapted for 10 min using white light of 30 cd s/m^2^ amplitude that saturates rod photoreceptors. After light adaptation, the photopic response was recorded to measure cone photoreceptor response. The curve fit was done using the Michaelis-Menten equation, and statistical analyses were performed using GraphPad Prism software.

### Quantitative real-time PCR

Total RNA was extracted from cells using TRIzol (Invitrogen). The reverse transcription reaction was conducted using SuperScript IV VILO master mix (Invitrogen). The quantitative real-time PCR experiments were performed using 2× Brilliant II SYBR-Green reagents (Agilent). Primers used for *HSP90aa1* are (5′-CCAGACCCATGCTAACAGGA-3′ and reverse primer, 5′- AGGGGAGGCATTTCTTCAGT-3′), *HSP90ab1* (5′-CAATGACTGGGAGGACCACT reverse primer, 5′-ACACGGCGGACATACAATTT-3′), *Pdeg* (5′-AAGCTAAGGGTCACTGCAGT-3′ and reverse primer, 5′-AAGGGCAGATGACGGTGATA-3′), PDE6A (5′-GCACGGCAAAGAAGACATCA-3′) and reverse primer, 5′-CATCCACCCAGACTCATCCA-3′) and *Pde6b* (5′-TGCTGACTGTGAGGAGGATG-3′ and reverse primer, 5′-CTTTCGGACTACACCCAGCT-3′, *Ywhaz* was used as a control, (5′-GTTGTAGGAGCCCGTAGGTCATCG-3′ and reverse primer, 5′-GCTTTCTGGTTGCGAAGCATTGGG-3′). Each sample was run in triplicate, and each gene was normalized to *Ywhaz*. The transcripts level of each gene in the HSP90α knockout (*Hsp90α*^*−/−*^) are represented relative to transcripts levels in control (*Hsp90α*^*+/+*^) group.

### Isobaric labeling TMT-based quantitative proteomics

Retinas from five HSP90α knockout and littermate wildtype animals were collected and flash frozen. Both males and females were used. The frozen samples were sent to IDEA national resources for proteomics analysis. Briefly, two retinas in a tube were lysed in 200 μl of RIPA buffer (Research Products International), followed by trypsin digestion using chloroform/methanol extraction. Tryptic peptides were labeled with Tandem mass tags (TMT; Thermo Scientific) using the 10-plex labeling kit. Labeled peptides were fractionated using high-pH peptide fractionation using 18 super-fractions. MS3 fragmentation of labeled peptides was done with a 60-min gradient per fraction using Orbitrap Eclipse acquisition. After the database search and bioinformatics analysis was performed using Scaffold 4 4.11.1. The data enrichment and profiling were analyzed using Metascape software.

### Protein deglycosylation assay

We used the Protein Deglycosylation Mix II kit (NEB, Catalog # P6044S) to analyze the status of glycosylation in Rhodopsin. The protocol provided by the manufacturer with slight modifications noted below was followed. The retina from *Hsp90α*^*+/+*^ and *Hsp90α*^*−/−*^ mice were collected at P15 and sonicated in the 200 μl lysis buffer (50 mM Tris, pH 7.4, 300 mM NaCl, 5 mM EDTA, and 0.02% Sodium Azide) containing protease inhibitors, 10 mM of iodoacetamide and 1% Triton X-100. Lysates were cleared by centrifugation at 14,000*g* for 10 min at 4 °C. 40 μl of lysate was combined with 2 μl of Glycoprotein Denaturing Buffer. After incubating at 75 °C for 10 min, 5 μl Protein Deglycosylation Mix II was added. The glycoprotein buffer was added in place of Deglycosylation Mix II, for the untreated control sample. The mix sample reaction was moved to 37 °C and incubated for 1 h. The reaction was terminated by cooling the mixture on ice and further analysis was performed by immunoblotting.

### Transmission electron microscopy

Ultrastructural analysis was done in the TEM core at West Virginia University. In brief, we collected mouse eyes, punctured a hole in the cornea, and incubated them in the TEM fixative (2% PFA, 2.5% glutaraldehyde (EMS), 100 Mm cacodylate, pH 7.4 (EMS-catalog: 11653) for 60 min at room temperature. After 1 h of fixation, the cornea and lens were removed, followed by further fixation at room temperature by gently rolling the vial for 2 days.

Fixed eye cups were dissected into small rectangle/trapezoid sections with the retina facing up. Retinal sections were washed in 0.1 M cacodylate buffer and incubated in 2% osmium tetroxide (EMS-catalog: 19170) prepared in 0.2 M cacodylate buffer, pH 7.4) for 1 h at 4 °C. We washed the retina in water (four times), followed by overnight incubation in 1% uranyl acetate. Retinas were dehydrated in an increasing ethanol series: 30%, 50%, 70%, and 95%. Retinas were dehydrated one more time in 100% ethanol, 2×, for 15 min. Dehydrated retinas were then washed in 2× propylene oxide, followed by incubated in a series of propylene oxide and resin mix as follows: 50%:50% (2 h), 25%:75% (overnight), and 100% resin, 2× at RT (2 h). Resin-embedded sections were then mounted in silicone EM molds and cured at a 60 °C oven overnight. Ultrathin resin sections of 50 to 100 nm were cut using Leica UC6 ultramicrotome with a Diatome diamond knife. Ultrathin sections were collected onto cleaned grids (formvar-coated or Athene). To stain the grids, we placed one drop of filtered Lead Citrate Reynolds Stain 3%, pH12 (EMS- catalog: 22410-01) on a strip of clean parafilm on a slide for 2 min. After the staining step, the grids were rinsed in filtered water and dried. TEM imaging was performed on a JEOL JEM-1010 at magnifications up to 20,000×. TEM images were further processed in Fiji/ImageJ.

### Tunel assay

Tunel assay was done using *In Situ* Cell Death Detection kit, fluorescein (Roche: 11684795910) following the manufacturer's instructions. Briefly, fixed retinal sections were washed two times with 1× PBS Followed by one wash in 1× PBS-T (0.01% Triton in 1× PBS), 10 min for permeabilization. After the PBS-T wash, the sections were incubated in 100ul of the Tunel stain for 1 h at 37 °C. For positive control, one section was treated with DNase recombinase I (Thermofisher scientist: FEREN0525) for 10 min before Tunel staining and the negative control was a section stained with the label solution lacking the tdt enzyme. The samples were kept in humidified and covered slide holder to avoid sample bleaching. After 1 h, samples were washed three times in 1× PBS. Tunel stain was co-stained with DAPI (Thermofisher scientist: 622480). DAPI antibody (1:2000) was added to the slide and incubated for 2 h followed by three washes with 1× PBS. ProLong gold (Invitrogen: P36934) was added to each section, cover slipped, and imaged using Nikon C2 confocal microscopy.

### Data and statistical analysis

All quantitative analyses were performed on age-matched knockouts and their littermate wild-type controls. Both sexes were considered for all experiments performed in mice, and no significant difference was observed. All data are a representation of a minimum of three independent experiments. Statistical analyses were performed using GraphPad Prism software version 7.0. Data are presented as mean ± SEM. Unpaired Student *t* tests were conducted to compare measured values between control and mutant samples. Scotopic and photopic ERG responses were analyzed with two-way ANOVA with a *p*-value of <0.05, and densitometry analysis was carried out using NIH ImageJ software. The figures were prepared using Adobe Illustrator software.

## Data availability

All the data supporting our findings are contained within the manuscript.

## Supporting information

This article contains supporting information.

## Conflict of interest

The authors declare that they have no conflicts of interest with the contents of this article.
