# Supplementary material for: HSP90α is needed for the survival of rod photoreceptors and regulates the expression of rod PDE6 subunits
Source: J Biol Chem. 2023 May 11;299(6):104809. doi: 10.1016/j.jbc.2023.104809 (PMC10250166; doi:10.1016/j.jbc.2023.104809)
Supplement: Supporting information [file mmc1.pdf]

HSP90α is needed for the survival of rod photoreceptors and regulates the expression of rod PDE6 subunits.

Daniella Munezero [1,2], Hunter Aliff [2,3], Ezequiel Salido [2,3], Thamaraiselvi Saravanan [2,3], Urikhan Sanzhaeva [2,3] , Tongju Guan [2,3] and Visvanathan Ramamurthy [1,2,3]*.

[1] Department of Pharmaceutical and Pharmacological Sciences, West Virginia University; Morgantown, West Virginia, USA, 26506.

[2] Ophthalmology and Visual Sciences, West Virginia University; Morgantown, West Virginia, USA, 26506.

[3] Biochemistry and Molecular Medicine, West Virginia University; Morgantown, West Virginia, USA, 26506.

*Corresponding author: Visvanathan Ramamurthy, Department of Biochemistry and Molecular Medicine, and Department of Ophthalmology and Visual Sciences, West Virginia University School of Medicine; 64 Medical Center Dr. Morgantown, WV, USA, 26506; Email: ramamurthyv@hsc.wvu.edu; Telephone: 304-216-2133; Fax: 304-293-6846.

List of materials included.

I. **Figure S1**: *Hsp90α*$^{-/-}$ males are infertile.

II. **Figure S2**: HSP90 distribution in the retina.

III. **Figure S3**: Sensitivity curve of scotopic and photopic responses in *Hsp90α*$^{-/-}$ and littermate control at different ages.

IV. **Figure S4**: HSP90α is expressed in cone photoreceptors in an all-cone retina mouse model.

V. **Figure S5**: Apoptosis and activation of immune response in the retina lacking HSP90α.

VI. **Figure S6**: Absence of HSP90α does not affect Golgi function, morphology, or Golgi-resident protein levels.

VII. **Figure S7**: Normal trafficking of photoreceptor proteins in HSP90α knockout.

VIII. **Figure S8**: Alteration in HSP90β levels is not linked to photoreceptor degeneration.

IX. **Figure S9:** Photoreceptor cell death in HSP90α knockout is not triggered by proteasomal insufficiency.

X. **Table. S1**: List of differentially expressed proteins in HSP90α knockout.

XI. **Table. S2**: List of antibodies used in the manuscript.

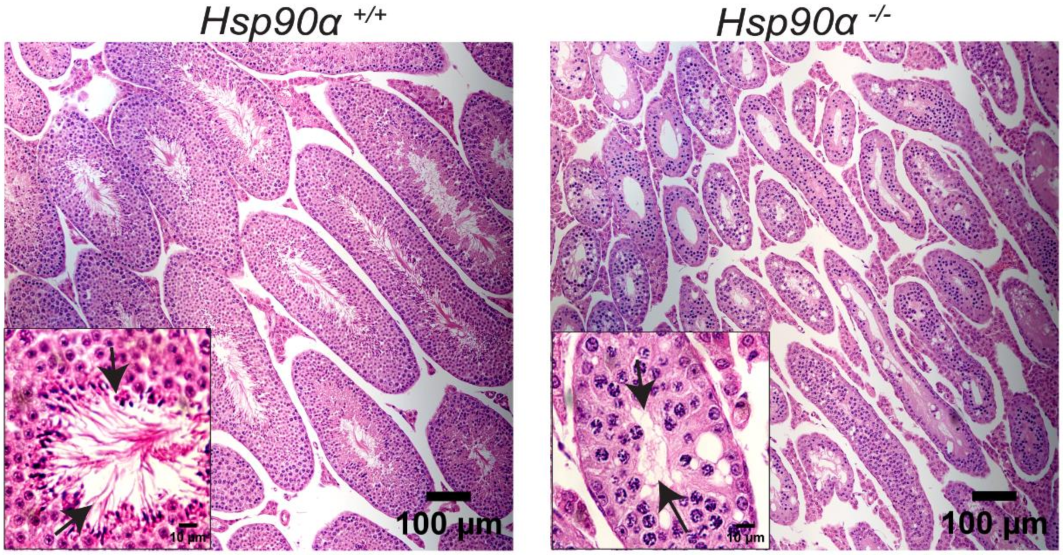

Supplemental Figure 1

Figure S1. ***Hsp90α*$^{-/-}$ males are infertile.** Sections from testis of wildtype (*Hsp90α* $^{+/+}$) and mutant (*Hsp90α*$^{-/-}$) mice at P115 stained with H&E. The insert shows seminiferous tubule, arrow points to the spermatozoa near the lumen in *Hsp90α* $^{+/+}$ and lack of spermatozoa in *Hsp90α*$^{-/-}$).

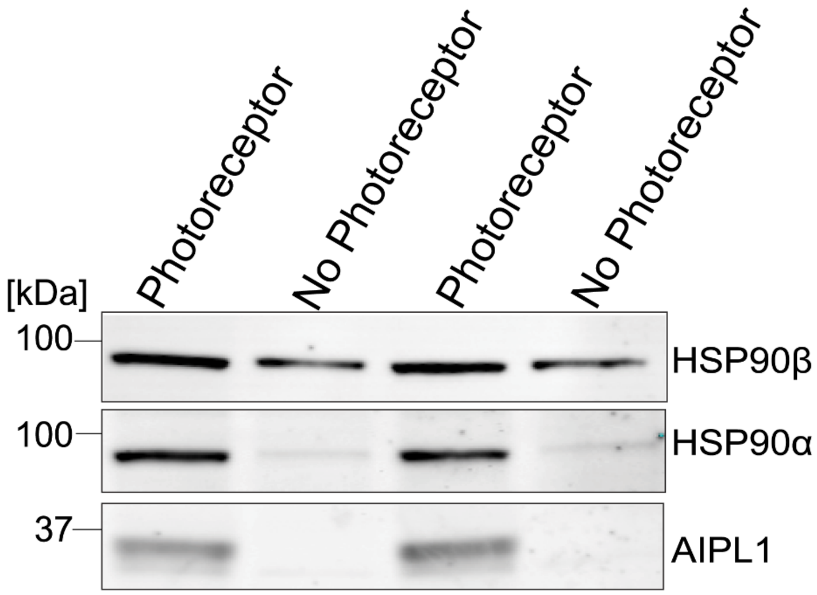

Supplemental Figure 2

Figure S2. **HSP90 distribution in the retina.**

Immunoblotting shows the levels of indicated proteins in wildtype retinas with intact photoreceptors (**Photoreceptor**) and in adult AIPL1 knockout retinas where photoreceptors have completely degenerated (**No photoreceptor).** Proteins assessed are indicated on the right. Molecular weight in Kilo Daltons (kDa) is indicated on the left.

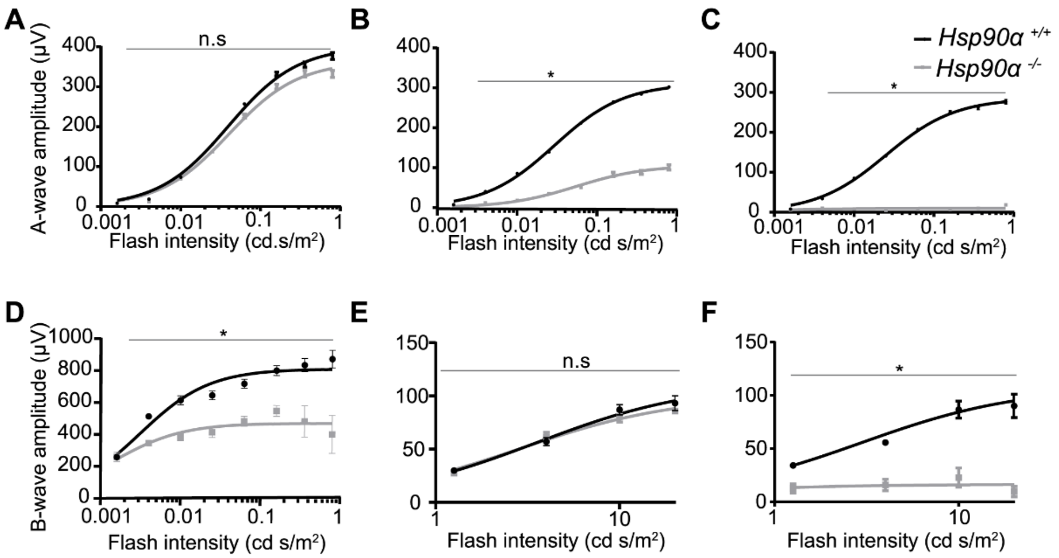

Supplemental Figure 3

Figure S3. **Sensitivity curve of scotopic and photopic responses in *Hsp90α*$^{-/-}$(black) and *Hsp90α* $^{+/+}$ control (gray) at different ages**. Varying light intensities ranging from 0.0016 to 20 cd.s/m^2 against corresponding responses were fitted using the Michaelis-Menten equation. Scotopic "a" wave response at postnatal day (P) 30 (***A***), P160 (***B***), and P250 (***C***). ***D,*** scotopic "b" wave at P30. ***E-F*** shows photopic "b" wave response at P160 (***E***) and P250 (***F***). Responses are depicted as Mean ± SEM of N = 6 eyes; Statistical analysis was performed using two-way ANOVA using Graph Pad Prism with n.s representing non-significant p-value (P) > 0.05 and an asterisk (*) indicating significant p-value (P) ≤ 0.05.

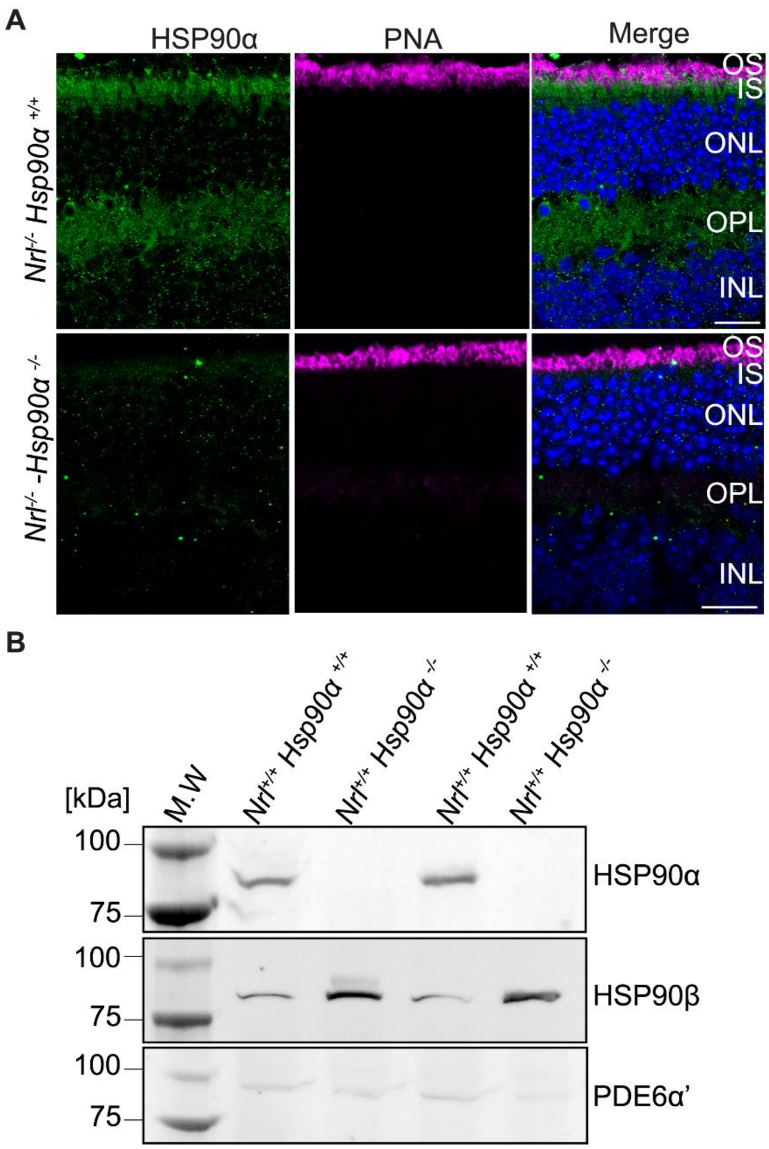

Supplemental Figure 4

Figure S4. HSP90α is expressed in cone photoreceptors in an all-cone retina mouse model.

A, immunofluorescence staining in retinal cross-sections from an all-cone retina (*Nrl*$^{-/-}$) and *Nrl*$^{-/-}$ *Hsp90α*$^{-/-}$ Sections were stained with HSP90α antibody and PNA that marks the cone sheath. The scale bar is 20 µm. OS: outer segment, IS: inner segment, ONL: outer nuclear layer, OPL: outer plexiform layer, INL: inner nuclear layer. ***B,*** immunoblotting of retinal extract derived from indicated animal models. Notice the increase in expression of HSP90β in the double knockout (*Nrl*$^{-/-}$*Hsp90α*$^{-/-}$). The antibodies used for probing the immunoblots are shown on the right. Molecular weight (M.W) in Kilo Daltons (kDa) is indicated on the left.

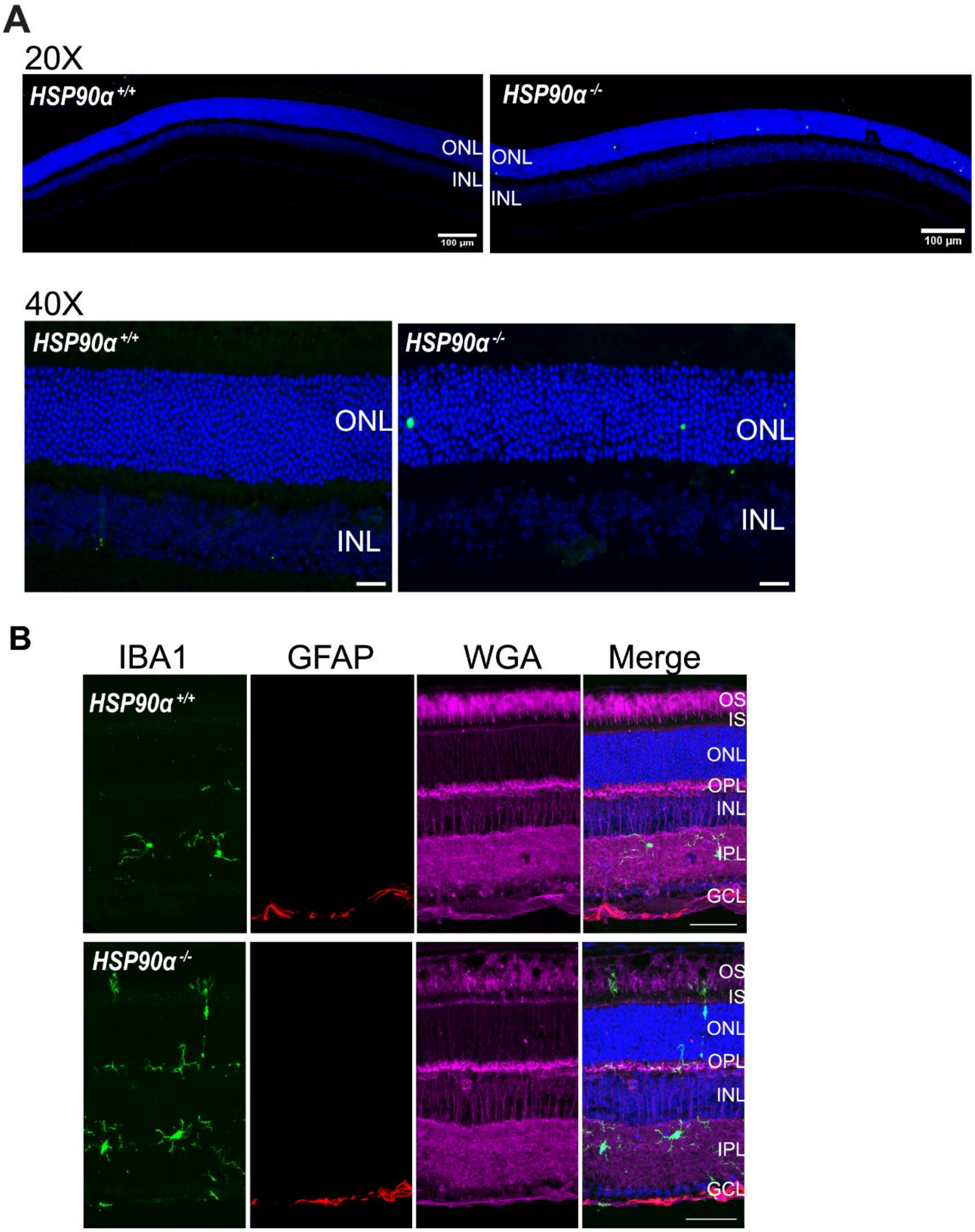

Supplemental Figure 5

Figure S5. Activation of immune response in the retina lacking HSP90α.

A*,** Retina lacking HSP90α undergoes apoptosis before the cell loss as illustrated by TUNEL-positive nuclei stained with DAPI in retinal sections at P45. wildtype (*HSP90α*$^{+/+}$*),* and knockout (*HSP90α* $^{-/-}$*)* retinal sections stained with TUNEL (green) co-stained with DAPI (blue). Top (20x) and bottom (40x, scale bar 20μm) magnification images of the Tunel staining are shown. ***B, immunohistochemistry on retinal cross-sections from P60 animals, stained with a microglia marker, IBA1 antibody (green), A Müller glia marker, GFAP (red), WGA (magenta), and nuclei marker, DAPI (blue). Both experiments are a representation of a minimum of three replicates from different mice. scale bar 20μm.

A

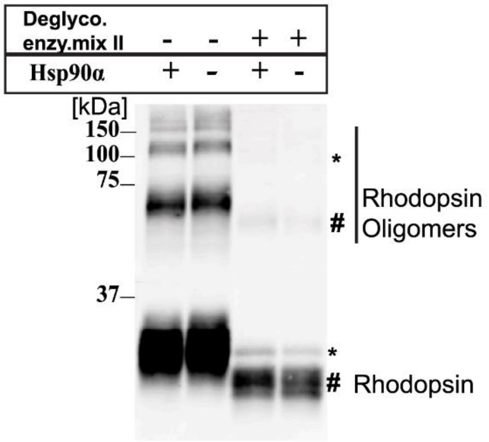

B

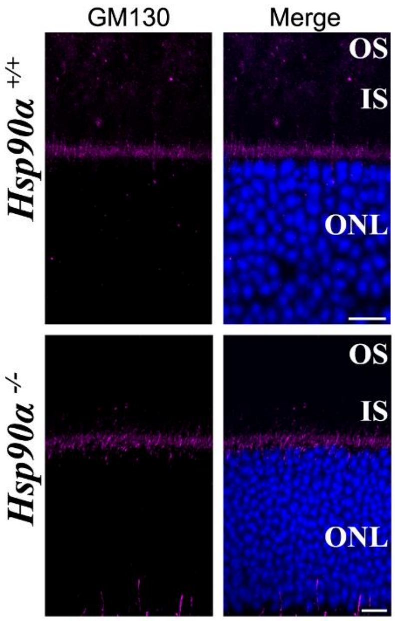

C

ID	Symbol	Log.FC	Adj. P values
Q8BMP6	Acbd3	0.06761	0.8979
Q8C7Y2	Cog6	0.02853	0.2897
A0A0R4J0Q9	Cog7	0.0722	0.9631
P55937	Golga3	0.06183	0.6418
P54797	Tango2	-0.03853	0.1453
Q9DC16	Ergic1	0.0254	0.1838
Q921M4	Golga2	0.09876	1.043
A2A9I0	Gosr2	-0.05162	0.2599
Q99JX3	Gorasp2	0.03196	0.2304
E9QL65	Cog3	0.1088	1.247
Q61543	Glg1	-0.008706	0.03723
Q8R088	Golph3l	0.06355	0.4588
Q8BH60	Gopc	0.02435	0.2197
O88630	Gosr1	0.1602	0.8858
Q921L5	Cog2	0.1284	2.636
Q91X51	Gorasp1	-0.02959	0.252
Q9Z160	Cog1	0.1078	1.506
Q9JJA2	Cog8	0.07345	0.7246
Q62313	Tgoln1	0.06693	0.5012
Q9CW79	Golga1	-0.007907	0.0798
Q8BRM2	Gorab	0.06514	0.425
Q8BS95	Gpr89a	0.05419	0.5072
Q8BXA1	Golim4	0.05328	0.7148
Q8C0L8	Cog5	0.05898	0.7677
Q8C3S2	Tango6	0.0214	0.1122
Q8R1U1	Cog4	0.05438	0.5658
Q91VW5	Golga4	0.05181	0.5068
Q91W53	Golga7	-0.0424	0.1522
Q9CQE7	Ergic3	0.007129	0.05021
Q9CR89	Ergic2	0.01053	0.06065
Q9CRA5	Golph3	0.07499	0.6192
Q9CYL5	Glipr2	-0.2279	1.129
Q9D1H7	Get4	-0.01603	0.1343
Q9D8T4	Tvp23b	0.1634	0.559
Q9QYE6	Golga5	0.1088	1.21
Q9QYR6	Map1a	0.01787	0.1204
P14873	Map1b	0.1587	1.913
Q9CQV6	Map1lc3b	0.08567	1.078
Q8C052	Map1s	0.1312	2.349
Q91VR7	Map1lc3a	0.157	2.348

Supplemental Figure 6

Figure S6: Absence of HSP90α does not affect Golgi function, morphology, or Golgi resident proteins levels.

A, Rhodopsin processing in the presence (+) and absence (-) of HSP90α. Retinal lysate from *Hsp90α*$^{+/+}$ and *Hsp90α* $^{-/-}$ were treated with Deglycosylation Enzyme Mix II (lanes 3 and 4). Deglycosylation buffer was used in place of the Deglycosylation Enzyme Mix II for the nontreated samples (lanes 1 and 2). Glycosylated (*) and non-glycosylated (#) rhodopsin staining is shown. Rhodopsin oligomers can be seen in higher molecular weights. Molecular weight is shown on the left in kDa. ***B,*** Immunohistochemistry showing the expression of GM130, a Golgi marker (magenta), and DAPI, a nuclei marker (blue) in retinal cross-sections from *Hsp90α*$^{+/+}$ and *Hsp90α* $^{-/-}$ animals. The scale bar is 20 μm. Each experiment was replicated three times. ***C,*** Golgi-specific proteins, and microtubule associated proteins (MAPs) changes in retinal samples from *Hsp90α*$^{+/+}$ and *Hsp90α* $^{-/-}$ animals quantified by TMT proteomics.

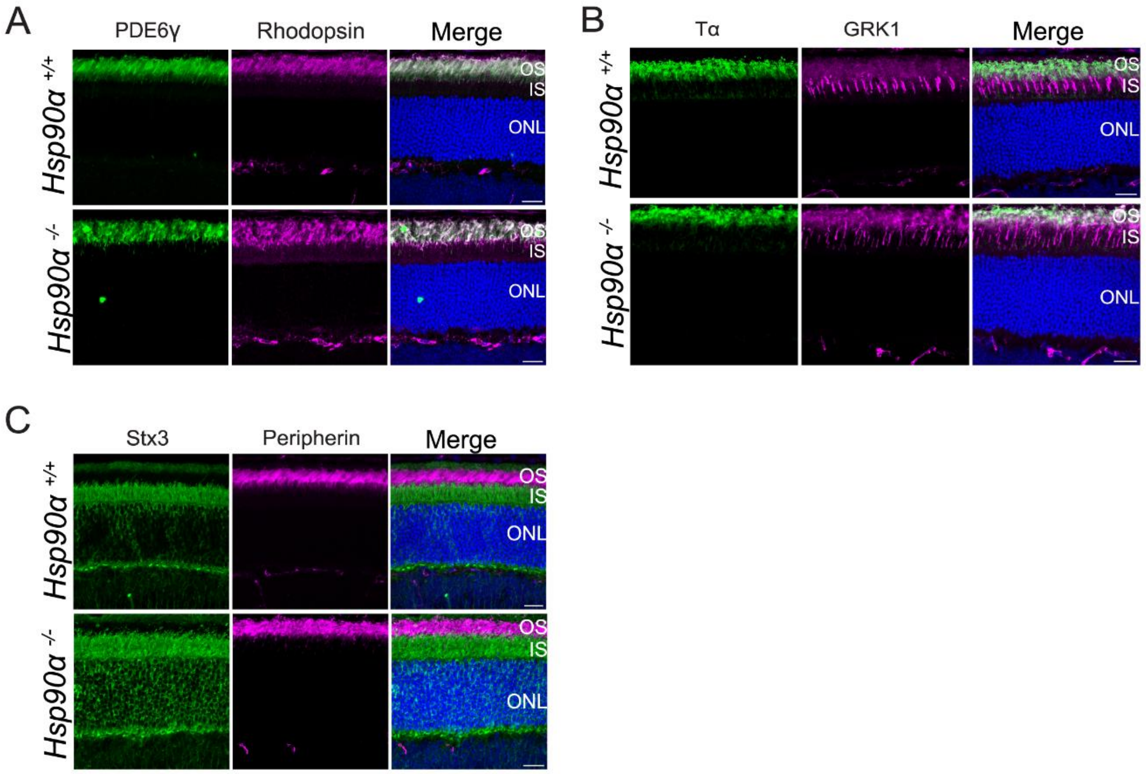

Supplemental Figure 7

Figure S7. Normal trafficking of photoreceptor proteins in HSP90α knockout.

Immunohistochemistry using retinal cross-sections obtained at P15 from $Hsp90\alpha^{+/+}$ and $Hsp90\alpha^{-/-}$ animals. Sections are stained with antibodies against different outer segment proteins. ***A,*** PDE6γ (green), counterstained with rhodopsin (magenta). ***B,*** rod-transducin (Tα) (green) and Rhodopsin kinase (GRK1) (magenta). ***C,*** Syntaxin 3 (Stx3) (green), and Peripherin (magenta). DAPI (blue) stains the nuclei. Merge shows all the channels together. OS: outer segment, IS: inner segment, ONL: outer nuclear layer. Scale bar = 20μm.

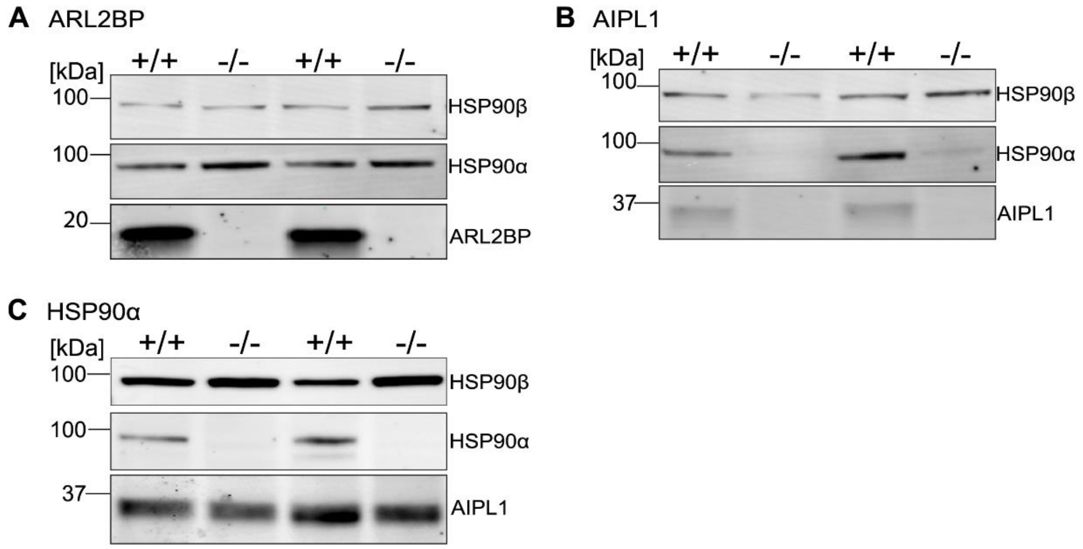

Supplemental Figure 8

Figure S8. **Alteration in HSP90β levels is not linked to photoreceptor degeneration**. Immunoblotting of retina extracts derived from indicated knockout animal models: ARL2BP, AIPL1, and HSP90α (-/-) and littermate wildtype controls (+/+). All the retinal extracts were collected from the corresponding knockout at post-natal day (P) P16 before photoreceptor degeneration had started. ***A,*** ARL2BP knockout. ***B,*** AIPL1 knockout. ***C,*** HSP90α knockout. The proteins assessed by immunoblotting are indicated on the right. The molecular weight in Kilo Daltons (kDa) is on the left.

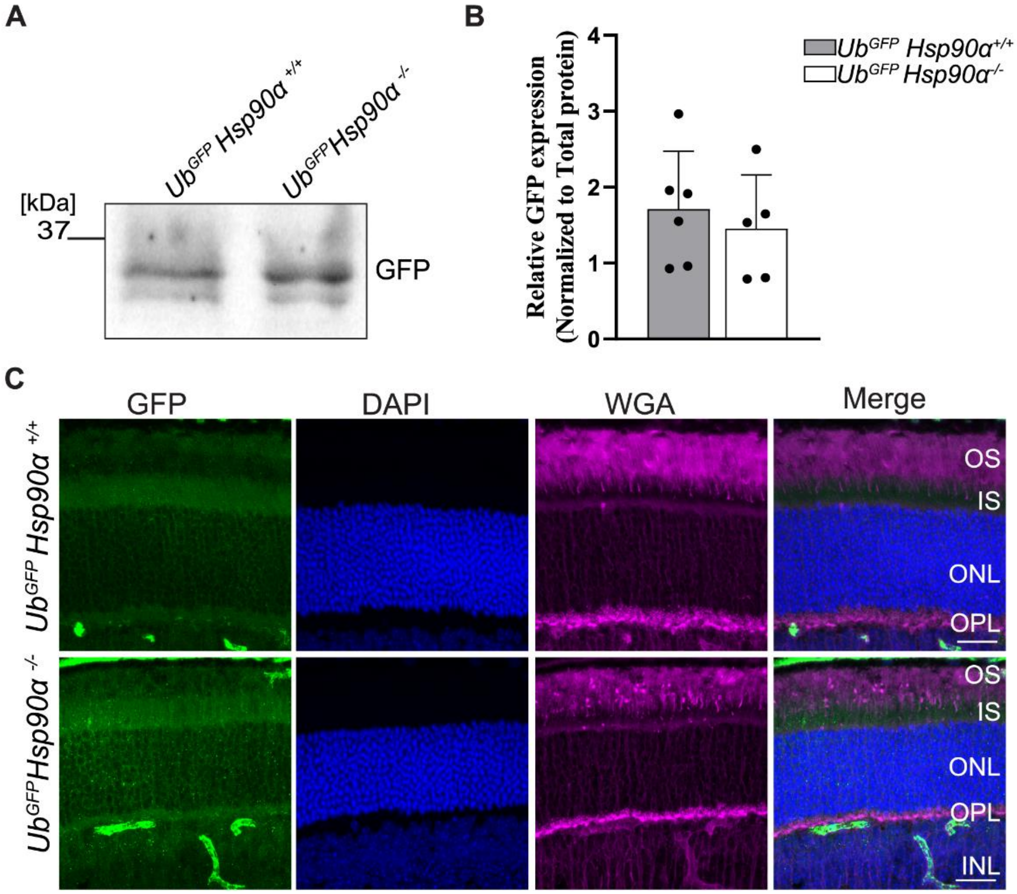

Supplemental Figure 9

Figure S9. **Photoreceptor cell death in HSP90α knockout is not triggered by proteasomal insufficiency.** ***A,*** immunoblotting of retinal extracts derived from indicated animal models at P45 stained with GFP antibody. The molecular weight in Kilo Daltons (kDa) is on the left. ***B,*** quantification of the GFP expression in the retinal extracts. ***C***,

GFP expression in retinal cross-sections from UB^{GFP} *Hsp90α* $^{+/+}$ and UB^{GFP} *Hsp90α* $^{-/-}$ animals at P45. GFP (green), DAPI (blue), WGA (magenta), and merge showing all channels. Scale bar = 20μm.

Table. S1: Differentially expressed proteins in quantitative TMT proteomic assessment between *Hsp90α*$^{+/+}$ and *Hsp90α* $^{-/-}$ retina extract. Significantly changed proteins are listed below with corresponding ID number, symbol, fold change (logarithmic scale), and the adjusted P values (Adj.P. Val). downregulated proteins are shown in regular font, and Upregulated proteins are shown in bold font.

Table. S2: List of antibodies, host, vendors, catalog number/RRID, and antibody dilutions used in this study.
